# Organ-on-a-chip meets artificial intelligence in drug evaluation

**DOI:** 10.7150/thno.87266

**Published:** 2023-08-15

**Authors:** Shiwen Deng, Caifeng Li, Junxian Cao, Zhao Cui, Jiang Du, Zheng Fu, Hongjun Yang, Peng Chen

**Affiliations:** 1 Beijing Key Laboratory of Traditional Chinese Medicine Basic Research on Prevention and Treatment for Major Diseases, Experimental Research Center, China Academy of Chinese Medical Sciences, Beijing 100700, China.; 2 Institute of Chinese Materia Medica, China Academy of Chinese Medical Sciences, Beijing 100700, China.; 3 Yunnan Biovalley Pharmaceutical Co., Ltd, Kunming 650503, China.; 4 Robot Intelligent Laboratory of Traditional Chinese Medicine, Experimental Research Center, China Academy of Chinese Medical Sciences & MEGAROBO, Beijing 100700, China.

**Keywords:** Organ-on-a-chip, Microfluidics, Drug evaluation, Artificial intelligence, *In vitro* model.

## Abstract

Drug evaluation has always been an important area of research in the pharmaceutical industry. However, animal welfare protection and other shortcomings of traditional drug development models pose obstacles and challenges to drug evaluation. Organ-on-a-chip (OoC) technology, which simulates human organs on a chip of the physiological environment and functionality, and with high fidelity reproduction organ-level of physiology or pathophysiology, exhibits great promise for innovating the drug development pipeline. Meanwhile, the advancement in artificial intelligence (AI) provides more improvements for the design and data processing of OoCs. Here, we review the current progress that has been made to generate OoC platforms, and how human single and multi-OoCs have been used in applications, including drug testing, disease modeling, and personalized medicine. Moreover, we discuss issues facing the field, such as large data processing and reproducibility, and point to the integration of OoCs and AI in data analysis and automation, which is of great benefit in future drug evaluation. Finally, we look forward to the opportunities and challenges faced by the coupling of OoCs and AI. In summary, advancements in OoCs development, and future combinations with AI, will eventually break the current state of drug evaluation.

## Introduction

Drug discovery and development is one of the most significant translational science activities contributing to human health and well-being. Nevertheless, the discovery and development pipelines are time-consuming and incur massive costs, primarily because of the preclinical validation as well as clinical trials involved 1, 2. It is estimated that over 10 years are needed to evaluate a new drug before it enters the market, and the average cost will be $2.5-5 billion 3, 4. Generally, a standard drug discovery process can be conceptually divided into three parts: target selection, lead identification, and preclinical studies 5. In the early preclinical stage of drug development, drug evaluation is crucial for confidently advancing a new drug candidate. Drug evaluation mainly focuses on physicochemical properties, biological activity, toxicity, safety, metabolism, pharmacological efficacy, and medicinal value of newly developed drugs, which in order to preliminarily verify their safety and effectiveness for further clinical trials, and to protect people from drugs which are unsafe, ineffective, or both 6-8. Traditional drug evaluation has mainly relied on cellular monolayer planar culture models and animal experiments. However, traditional methods face several challenges, in part due to the intrinsic limitations of two-dimensional (2D) cell culture models that may not be able to mimic the microenvironment in an organ, and animal models may not accurately represent what occurs in humans 9-11. In addition, animal models are often not suitable for high-throughput bioassays as well as large-scale drug screening 12, and are also often cost-prohibitive. A bill signed in December 2022 allows the United States Food and Drug Administration (FDA) to approve new drugs without being tested on animals. This marks a major change in people's use of animals after more than 80 years of drug safety supervision. Thus, it necessitates quick and robust methods with the goal of discovering, analyzing, and optimizing a reliable drug candidate 1, 13.

Microfluidics is the science and technology of manipulating and detecting fluids on a micro-scale 14. With its obvious advantages, including fast processing speed, high spatial resolution, sensitivity, and integration, easy control, and low cost of reagents, microfluidics has become an increasingly attractive tool for both fundamental and practical research 15. Furthermore, microfluidics has already been utilized to create more *in vivo*-like models of cell culture because of the dimensional comparisons with biological cells 16, 17. Notably, microfluidics has the ability to capture, align, and manipulate single cells in drug discovery. Furthermore, microfluidic system has the capability for higher-throughput screening, and it could be used for screening drugs at different species and concentrations. As a valuable tool for developing more *in vitro* models which capture cellular and organ-level responses, microfluidic technology is widely used for fast and animal-free risk evaluation of new drugs 18.

As a product of microfluidic technology gradually developed, OoCs could faithfully mimic the pathophysiological microenvironment of target organs* in vivo*, offering exciting potential to bridge the gap between *in vitro* evaluation models and *in vivo* pathophysiological complexity 19, 20. In 2004, adapting microfluidic technology for modeling organs and systemic-level functions of human physiology or disease research was first published 21. Then, the most famous and landmark OoC device, known as the 'breathing lung' (lung-on-a-chip) was designed in 2010 22, which initiated the advancement of the biologically inspired OoCs today. Since then, examples of single OoCs include brain/blood-brain barrier 23-25, lung 22, 26, 27, heart 28-30, liver 31-33, kidney 34-36, gut 37-39, vasculature 40-42, skin 43, 44, bone/bone marrow 45, 46, retina 47, 48, muscle 49, 50, fat 51, 52, and tumor/cancer 53-56 have been successfully developed, all of these can be used for drug research. Furthermore, it is possible to investigate organ-organ interactions and systemic diseases like drug off-target toxicity, cancer metastasis, and inflammation by coupling multiple OoC platforms together through vascular perfusion of supernatant exchange or a shared blood substitute 57.

The mechanism of action of drugs is diverse, with various phenotypic effects on cells and organs. Simply recognizing and categorizing these features from the perspective of molecular indicator detection is time-consuming and laborious, which has become a challenge for large-scale molecular library (estimated to be more than 10^60^ molecules 58) drug screening, and it is even more difficult to display real-time changes in cellular mechanisms. Nowadays, the functional disclosure of drug targets tends to reveal their functions in the dynamic process of life. During this process, drug evaluation with the OoC platform will generate many images and datasets, and the feature extraction of these dynamic data cannot be completed manually. In recent years, the application of AI in microfluidics has achieved significant results, with new deep learning methods and deep neural network models constantly emerging. OoCs are now starting to attract AI, especially the machine learning (ML) and deep learning (DL) approaches to experimental design and data interpretation 57. Deep learning was introduced into the field of machine learning by Rina Dechter as early as 1986, and in 2000, Aizenberg introduced Artificial Neural Networks in the field of machine learning 59. Visual recognition and data processing based on AI will bring possibilities to solve the above problems, including culture conditions optimization, image detection and tracking, and processing such a large volume of data.

**Figure 1:**
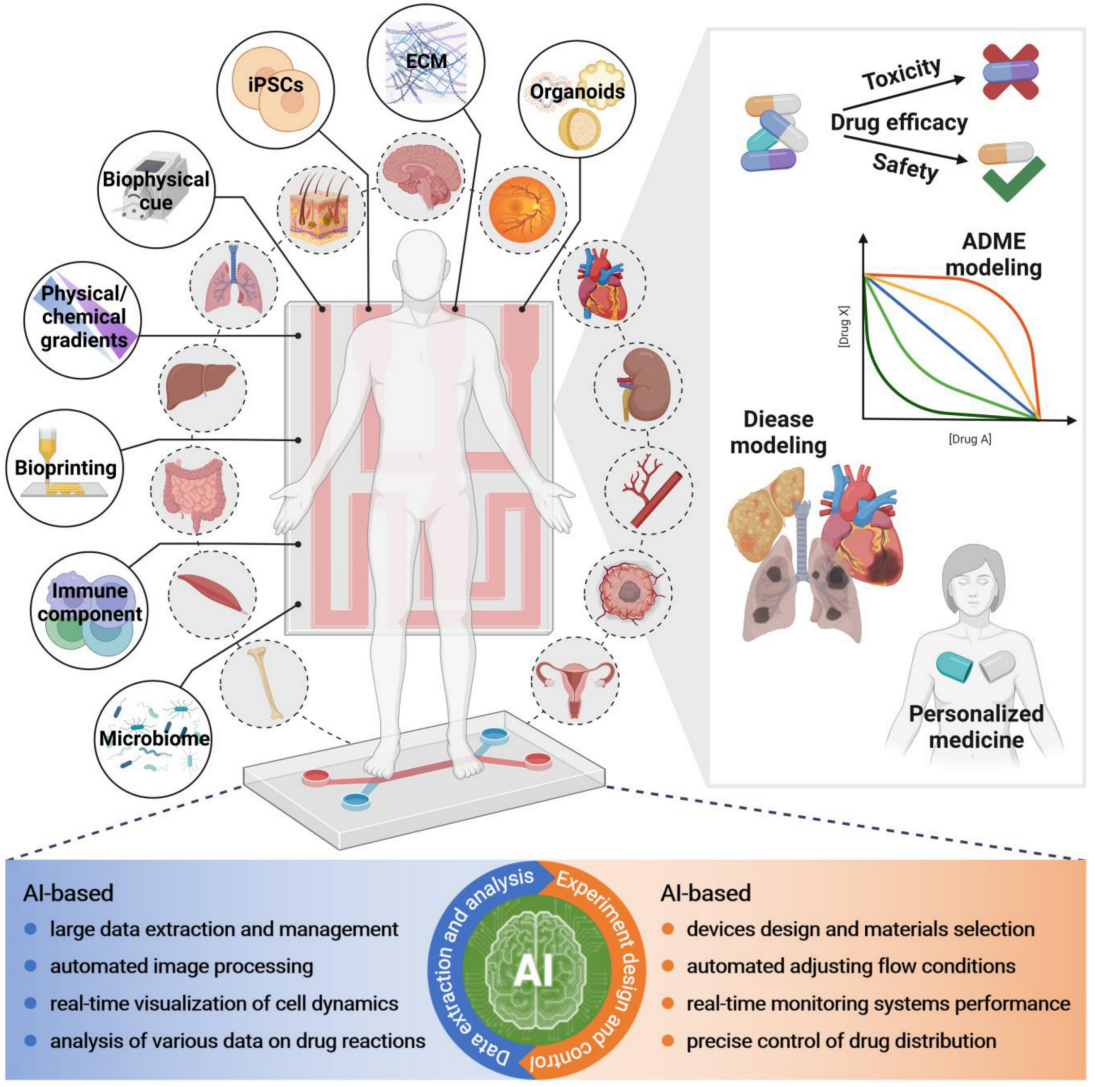
** Schematic of organ-on-a-chip meets artificial intelligence in drug evaluation.** OoCs have been utilized to model almost all organs in humans for drug testing, disease modeling, personalized medicine, and others. To improve the physiological relevance of OoCs, various factors including cell types, stimulations, and materials are considered and incorporated. Finally, OoCs combine with AI will be of great benefit in experiment design and control as well as data extraction and analysis, which holds exciting promise for drug evaluation with OoCs. Abbreviations: iPSCs: induced pluripotent stem cells; ECM: extracellular matrix. Created with BioRender (www.biorender.com).

At present, OoCs and AI are hot topics in research, and researchers hope to generate more possibilities through the combination of the two. Drug discovery and pharmacological researchers also hope to see this type of review article to obtain relevant knowledge simply and directly. However, most of the current reviews are still focused on discussing the combination of microfluidics and AI (machine learning and deep learning) 60-64. Although a recent review focused on the combination of OoCs and deep learning, the core of this review was not specifically on the application of integration in drug evaluation 19. In fact, drug evaluation is one of the most important areas of OoCs application. Thus, a summary of the application of OoCs in drug evaluation, as well as a timely and comprehensive review of the driving role of AI in this field, will facilitate the combination of both for drug evaluation in the future. In this review, we first give a brief overview of basic information on microfluidics-based organ-on-a-chip. Then, we introduce the most recent advances in the field of OoCs, which exhibit clinical mimicry by simulating human patient responses or have utilized this technology to further drug development and personalized medicine. Moreover, we reviewed typical cases of AI application in drug evaluation using OoCs, which will pave the way for future drug development (Figure 1). Finally, we discuss opportunities and challenges for the future of the field. In addition to cells and tissues, the “organ” here also includes organoids, and organoid-on-a-chip has been included in here OoCs.

## The background of OoCs invention

In traditional, 2D cultures cells grow in a culture flask or dish as an adherent monolayer, attached to a plastic surface 65. Although 2D monolayer-based assays have proven to be a valuable method for cell-based studies of low cost, ease to use, and high throughput, adherent culture also has numerous disadvantages, and its limitations have been increasingly recognized 66, 67. One such key limitation is that 2D cultured cells fail to accurately reproduce the natural human physiology, which prevents this culture method from replicating the cell-cell and cell-environment interactions present in native tissue. As a result, drugs respond differently between cells cultured in 2D and corresponding tissues 68. In addition, a drawback is that the cells in the monolayer have unrestricted access to the components of the medium, such as nutrients, metabolites, oxygen, and signal molecules 69. Meanwhile, adherent culture usually allows the study of only a single cell type, which results in cells lacking the microenvironment, or niches, in which they reside *in vivo*. Thus, the predictive value of 2D monoculture models is quite limited.

Efforts to address some limitations of 2D culture models, 3D culture models have been developed, which provide *in vivo*-like microenvironments and have received much attention. These models use synthetic or natural cell scaffolds (decellularized) to support cell attachment, growth, and morphogenesis in a 3D environment 70. Synthetic cell scaffolds typically contain biocompatible polymer materials, such as a variety of fiber and hydrogel scaffolds 71. Natural cell scaffolds are made from extracellular matrix gels, which contain such as collagen and glycoproteins, and minerals including hydroxyapatite 72. While the 3D conditions more closely resemble the *in vivo* state, these models remain lack the multiscale structures and tissue interfaces that are meaningful for organ function. In addition, the lack of controlled and precise application of nutrient supply gradients and chemical cues, results in the poor modeling of an *in vivo* physiological microenvironment. Importantly, cells are typically not exposed to physical stimuli that are essential for organ development and functioning 6. Of note, one of the most significant paradigm changes in medicine recently has been the recognition of the central role displayed by the microbiome, which is made up of host-specific communities of commensal microbes, in human health and disease 39. However, it is not yet possible for human cells to co-culture with complex microbial that come into direct contact, as this frequently leads to culture contamination and cell death within hours 73. These all limit their use in drug screening.

Preclinical animal models are an essential component of the drug discovery and development process. Although animal models have offered a living system to assess the efficacy of drugs on target site and non-target organ toxicity, it captures the physiological complexity with a high degree of fidelity. However, it is not really representative of human physiology, pathological, and genetic characteristics, thus failing to accurately anticipate drug response in humans 74, as the pharmaceutical industry is gradually discovering. Of note, recent systematic studies on the correlation between animal data and human outcomes have shown a weak predictive ability of animal models 75, and the clinical translatability of drug efficacy tests conducted on animal models is highly controversial 76. Furthermore, animal models have been associated with ethical concerns, high costs, and low yields, as well as difficulty in performing high-throughput evaluations of drugs. Thus, preclinical drug testing models with better physiologically relevant are needed to simulate complex human-relevant conditions, enable high-throughput assessment of drug candidates, improve the success of clinical trials, and ultimately deliver safe and effective drugs to the market.

Organ-on-a-chip is an *in vitro* microphysiological system (MPS) used for mimicking the human body environment, representing a simplified but realistic model of its organ-level and even organism-level functional counterpart with functionality read-outs matching the intended application 77. Microfluidics-based OoCs take advantage of control strategies and multiparametric approaches designed for microfluidics, compared to static culture models, which allows better oxygen perfusion, continuous nutrient exchange, physiological microenvironments, and tissue mechanical forces to provide sufficient nutrients and necessary chemical/mechanical stimuli to better emulation of conditions within the organisms 78, 79. Notably, OoCs have realized co-culture with microorganisms 39, 80-82. Animal models often lack the ability to predict results in human drug response. Humans and animals differ substantially in physiological structure, complexity, tissue/organ function, and other parameters, resulting in reduced accuracy and reproducibility of experimental results 83. For instance, drug metabolism can lead to the production of metabolites with physicochemical and pharmacological properties significantly different from the parent drug, thereby enhancing biological activity or producing adverse biological consequences 84, 85. Thus, species differences in metabolism may result in an inability to predict the efficacy/toxicity of a drug in humans. For the same drug, it may have different or even opposite pharmacological effects between humans and animals due to differences in the species' target expression, binding capacity, and drug pharmacokinetics and pharmacodynamics (PK/PD). Furthermore, other problems such as ethical concerns, which have also greatly limited progress in drug development. To that end, as an emerging* in vitro* model, OoCs have been envisioned to replace animal studies. Meanwhile, OoCs may improve the current lack of female individuals in human clinical trials 73.

As a type of microfluidic device, OoCs are created with microchip-manufacturing methods with a miniaturization feature. Owing to the intrinsic characteristics of microfluidics (e.g., compact microchannels), OoCs can provide accurate control of biophysical, biochemical, and cellular parameters 86, and reduces the sample sizes and materials consumption required for drug testing 65. Importantly, OoCs can simulate chemical concentration gradients, which are essential for the regulation of various biological processes and drug studies. Furthermore, OoCs with a physiological barrier function can better mimic the delivery and absorption of drug compounds *in vivo* 87. Polydimethylsiloxane (PDMS) is the preferred choice for manufacturing OoCs, with advantages such as ease of fabrication and handling, gas permeability, low cost, and optical transparency for real-time culture monitoring 88. Finally, membranes can be integrated into the chips to create multiple channels and separate cells 65.

To date, researchers have developed single-organ-on-a-chip for almost organs in the human body, all of which can be used for drug research. Nevertheless, they lack both a systemic dimension and cross-organ communication 89. As the human body is a physiologically complicated system, thus necessary to evaluate drug disposition throughout the whole body as well as to quantify PK/PD parameters that contribute to direct clinical trial design, and try to gain more understanding of diseases which is caused by multiorgan interactions 90, 91. Multi-organ-on-a-chip, coupled single organs by flow, have been created to recapitulate organ-organ interactions and potentially whole-body responses to drugs and to serve as models for diseases 92.

**Figure 2:**
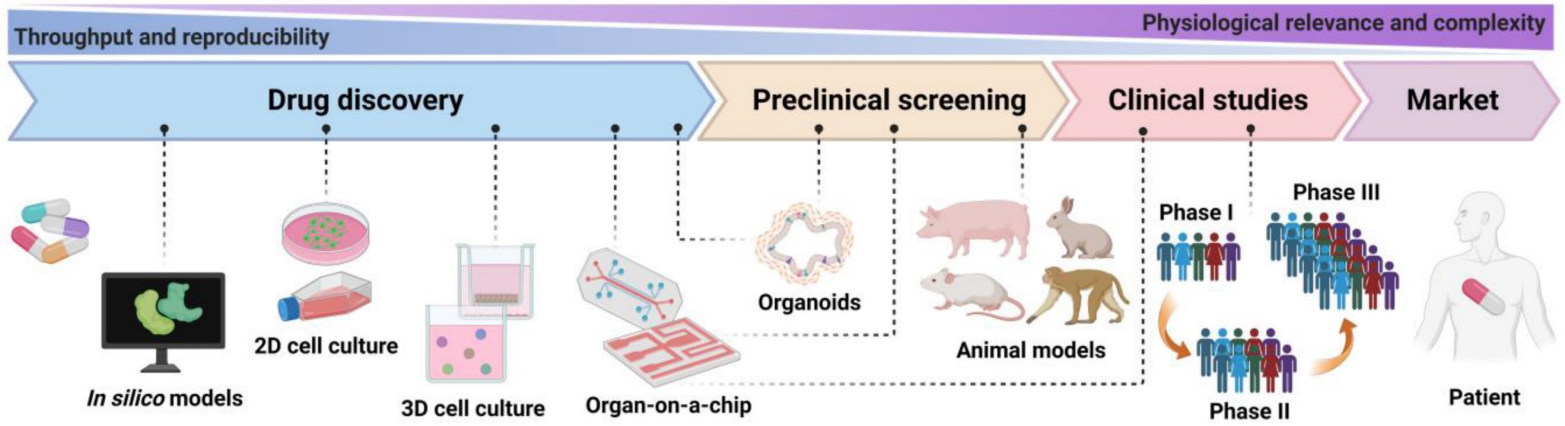
At various phases of drug development, the comparison of throughput and reproducibility with physiological relevance and complexity of different *in vitro* drug evaluation models. Created with BioRender (www.biorender.com).

After rapid developments in recent years, OoCs that replicate human organ functions are a promising technology for drug evaluation (e.g., drug transport, metabolism, toxicity, and therapeutic effects), disease modeling, and personalized medicine, which indicates its potential role in all phases of the drug development (Figure 2). The rise of OoCs has brought a new dawn to drug evaluation. Therefore, in the following, we will outline instances of various single and multi-OoCs examples to discuss recent advances in OoCs development, with a focus on their application in drug evaluation in a human-relevant manner.

## The application of single-organ-on-a-chip in drug evaluation

**Figure 3:**
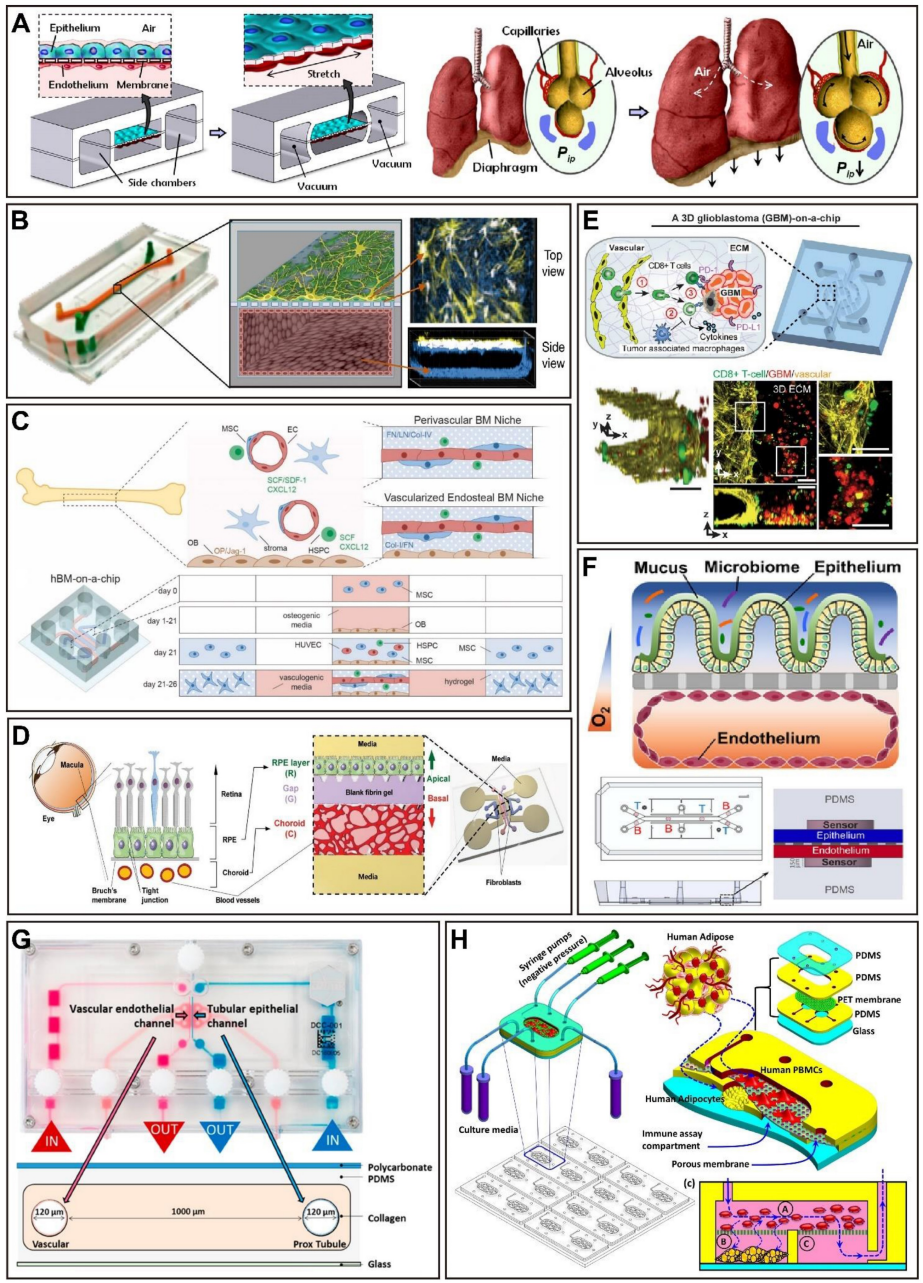
** Representative examples of single OoCs. (A)** An OoC platform simulates physiological breathing movements by applying a vacuum to the lateral chambers to produce mechanical stretching of the PDMS membrane that forms the alveolar-capillary barrier. Adapted with permission from 22, copyright 2010 American Association for the Advancement of Science. **(B)** Reconstitution of BBB in a microfluidic device showed that hypoxia-enhanced BBB OoC platform reproduces the barrier function and outlines the shuttling of drugs and antibodies. Adapted with permission from 23, copyright 2019 Nature Publishing Group. **(C)** A bone marrow (BM) OoC platform could summarize both the central perivascular BM niche (without OBs) and the vascularized endosteal BM niche (with OBs) that is discovered in the cavities of long bones. Adapted with permission from 46, copyright 2021 Elsevier. **(D)** A retina microfluidic platform including an RPE monolayer and adjacent perfusable blood vessel network with barrier function of oBRB successfully mimics the pathogenesis of CNV, especially in terms of morphogenesis. Adapted with permission from 48, copyright 2018 Wiley. **(E)** A patient-specific glioblastoma OoC platform with immunosuppressive tumor microenvironments was used to dissect the heterogeneity of immunosuppressive tumor microenvironments to optimize PD-1 immunotherapy. Adapted with permission from 54, copyright 2020 eLife Sciences Publications. **(F)** A gut OoC device contains a complex human microbiome, which makes the study of host-microbiome interactions possible. Adapted with permission from 39, copyright 2019 Springer Nature. **(G)** A vascularized dual-channel microphysiological system provides a platform to evaluate the renal secretion of novel drug candidates. Adapted with permission from 128, copyright 2020 American Chemical Society. **(H)** A fat OoC platform combines adipocyte and immune cells to model the inflamed adipose tissue for the analysis of immune-metabolic in type II diabetes. Adapted with permission from 51, copyright 2019 Nature Publishing Group.

The design guidelines for OoCs are founded on the objective of recapitulating the physiology of the organ system under study. Ideally, the OoCs environment should be created using a minimally functional (simplest feasible) unit of each organ system 78. Since 2010, almost all organ systems have been modeled using OoCs to gain a new understanding of the molecular and cellular underpinnings of various physiological and pathophysiological processes, and to recapitulate clinical responses to therapeutics seen in human patients 73. In this section, we review the key human single-organ-on-a-chip studies, especially under the background of drug development (Figure 3 and Table 1).

### Brain/BBB-on-a-chip

The structural and functional complexity of the human brain presents unique challenges for neurological drug development. A major obstacle is the blood-brain barrier (BBB), which selectively controls the passage of drugs into the central nervous system (CNS) and prevents it from blood-borne neurotoxic substances as well as maintains homeostasis for optimal brain function 93. In addition, the complexity also makes it challenging to research in non-human models. In this context, OoCs emulating the function of BBB is of particular interest as they enable testing of whether drugs used for the treatment of neuro-related diseases could act across the BBB to their designated targets 78, 94. The very first BBB model design consisted of an upper and a lower PDMS channel divided by a porous membrane, similar to a sandwich structure 95. Usually, astrocytes, pericytes, or other types of brain cells are cultured in the lower channel, while endothelial cells are seeded in the upper channel. Moreover, the neurovascular unit OoC systems were created to develop a more faithful model of the BBB 96, as the BBB is a significant obstacle to the delivery of a lot of neuroactive therapeutics. Although sometimes used interchangeably, the BBB is described as the neurovascular unit free of microglial and neuronal components 97. These models employ transendothelial resistance (TEER) as a functional readout, which is a gold standard method for measuring the 'tightness' of the constructed BBB 94. Previously, it has been demonstrated that using microfluidic perfusion improves physiological barrier function and offers a more predictive drug reaction 98. For instance, hypoxia-enhanced BBB OoC platform outlines the shuttling of CNS-targeting drugs and antibodies *in vivo*, which may contribute to the development of drugs or delivery vehicles (Figure 3B) 23. More recently, a BBB OoC device was employed to investigate stem cell-based therapies' therapeutic potential for ischemic stroke. This model demonstrated clinically relevant responses to an ischemic injury, and recapitulated the interactions between therapeutic stem cells and host cells 24. Therefore, a human-specific model of the BBB would enhance the comprehension of human neurodegenerative diseases and the discovery of neurological drugs.

### Lung-on-a-chip

As the lung fills with air, the respiratory regions cyclically expand and contract to increase the surface area accessible for gas exchange. When the alveoli were considered the smallest functional unit of the lung, cyclic expansion can be simulated by applying mechanical stretch to the gas exchange surface 78. The most well-known organ-on-a-chip, known as the 'breathing lung' (lung-on-a-chip) was designed in 2010 (Figure 3A) 22. This device has a microporous membrane between two layers of a channel construction which in human alveolar epithelial cells lined the upper layer of the membrane and human pulmonary endothelial cells lined the bottom layer. Once the alveolar cells were confluent, the medium inhaled from the upper channel formed an air-liquid interface with the alveolar cells. The lung structure is replicated on a platform using flowing air and culture medium, respectively, and the extension and contraction of the porous membrane are achieved by varying the internal pressure of the channels on either side of the channel during particular cycles to mimic physiological respiration 70, 99, 100. The subsequent model used a similar chip design and cell seeding with modifications and additional improvements for various applications, including replicating the drug toxicity seen in cancer patients receiving IL-2 26, and investigating the pulmonary toxicity of nanoparticles 101. In addition, the model of lung airway OoCs was designed to reproduce the lung airway microenvironment 102. Taking the presently well-known COVID-19 as an example, the lung airway OoC system was rapidly being used to repurpose FDA-approved drugs as possible treatments against SARS-CoV-2 103, and amodiaquine was discovered through this platform to be a potential entry inhibitor for SARS-CoV-2 27. More recently, a model that simulates alveoli *in vivo* using collagen and elastin has been developed, which was called the second-generation lung OoCs 104.

### Heart-on-a-chip

The heart is one of the least regenerative organs in the body 105, which is also a significant target organ for toxicity. Cardiotoxicity as one of the most common causes of drug failures 106, drives the development of heart OoCs. Cardiac muscle is a highly ordered dense tissue that is susceptible to interference from drugs, drug-drug interactions, or off-target side effects 79. So far, a variety of heart OoC platforms have been developed, including co-culture of multiple cell types such as cardiomyocytes, endothelial cells, and cardiac fibroblasts, focused on establishing biomimetic and functional aspects of the heart 107. Interestingly, most cardiac OoCs are primarily used in cardiotoxicity research. In order to improve the assembly of functional tissue models, anchoring pillars, posts, and wires were utilized to stretch cardiac tissues 108. A platform that used a 'Biowire' model showed it enabled the generation of highly aligned heart tissues and matured these microtissues by electrical stimulation to achieve functional characteristics resembling those of native human cardiac muscle 109. In addition, a novel based on 3D bioprinting was used to construct endothelialized human myocardium for cardiovascular toxicity evaluation, reproducing the cancer drug doxorubicin-related myocardial toxicity that has been clinically observed 28. However, a challenge is the limited ability of mature cardiomyocytes to self-renew 110. In this framework, induced pluripotent stem cells-derived cardiomyocytes (iPSC-CMs) hold great promise; yet, the limitation of its immaturity still remains, which ultimately affects the pharmacological response 28, 107. To obtain a cardiac model with adult-like features, methods such as mechanical, electrical, and hydrodynamic stimulation were used to improve tissue maturation 108. Despite this, it still further expands the potential application of heart OoCs in the field of cardiotoxicity. Heart OoCs are also used to evaluate potential treatments for COVID-19. A study found that azithromycin and hydroxychloroquine, two drugs considered to have therapeutic promise for SARS-CoV-2, when used separately or together as a therapy both have a proarrhythmic potential, which is in accordance with clinical literature 29. Another comparable study came to a similar conclusion 111.

### Liver-on-a-chip

The liver has a complex microarchitecture with various functions and displays a central role in the synthesis and metabolism of various substances 112. Drug-induced liver injury (DILI) is the most frequent reason for drug candidate failure in preclinical and clinical trials, as well as a common reason for withdrawal from the market after drug approval 113. Thus, during the drug discovery process, accurate prediction of its metabolic capacity and toxicity is extremely important 99. Today, a variety of *in vitro* models have already been developed to accurately mimic the complex liver architecture and physiology, and to generalize the human liver's response to drugs 114. Notably, these liver organoids successfully reproduce express cytochrome P450 and secrete serum albumin of hepatocytes, recapitulating the function of the native liver 115. The liver is constituted of approximately 1 million lobules which are its constitutional unit, and contain the hepatocytes responsible for drug metabolism 116. However, liver OoC systems usually use primary human hepatocytes or cell lines that decline in function with increasing culture time, which challenge could be overcome by co-cultures with like Kupffer cells, fibroblasts, stellate cells, and endothelial cells, as well as perfusion 88. Recently, a high-throughput hepatotoxicity screening OoC device, OrganoPlate LiverTox™, which contains iPSC-derived hepatocytes, endothelial cells, and Kupffer cells, was used to evaluate 159 compounds known to cause hepatotoxicity, and the toxicological prioritization scores were computed (Figure 5A) 31. Another collagen-based liver OoC platform showed better predictive sensitivity than all previously reported *in vitro* models after screening 122 clinical drugs for liver toxicity 117. Furthermore, a study that investigated the impacts of human population variability on liver drug metabolism with the use of hepatocytes from different donors, and an analysis of six drugs confirmed significant inter-donor variability in hepatocyte function. The predicted clearance values and those observed *in vivo* had excellent correlations 118. Of note, one of the beneficial applications of liver OoCs is to mimic human-specific hepatotoxicities, which is frequently overlooked in preclinical animal models 73. A study comparing human, dog, and rat liver OoC platform highlighting demonstrated species-specific differences in drug metabolism and toxicity (including hepatocellular injury, steatosis, cholestasis, and fibrosis) 33, showing the significance of employing human-specific cells in some experiments, while confirming the relevance of using non-human models. Meanwhile, the largest OoCs study to date, in which 780 liver OoC devices were used to evaluate the toxicity risk of a blinded group of 27 known hepatotoxic and nontoxic drugs, showed a sensitivity and specificity of 87% and 100% for liver OoCs, respectively 119. These results are superior to animal and microsphere models, and support the application of OoCs in preclinical toxicology evaluation. With further development, liver OoCs will contribute to predicting drug toxicity early and reduce the occurrence of adverse drug events.

### Kidney-on-a-chip

The kidney is a significant organ responsible for metabolism, excretion, and reabsorption, which is a frequent site of toxicity during drug discovery 120. Drug-induced kidney injury (DIKI) is frequently observed in drug therapy and may as a dose-limiting factor 121. Accurately identifying nephrotoxic compounds during the preclinical testing stage would enable effectively avoiding nephrotoxic drugs during development. The minimal functional unit of the kidney is the nephron, which contains the glomerulus, proximal convoluted tubule, loop of Henle, distal convoluted tubule, and collecting duct 122, 123. In 2008, a nephron-on-a-chip containing the glomerulus, the proximal tubule, and the loop of Henle was designed to replicate the function of a single nephron 124. Because of their physiological functions and high energy requirements, proximal tubule cells are particularly susceptible to drug toxicity 125. The first nephrotoxicity study was performed using proximal tubule OoC device consisting of Human Renal Proximal Tubular Epithelial Cells (HRPTEpiC), exposed to fluid flow 34, 126. After that, the proximal tubular model is the main type of OoCs to predict drug-induced nephrotoxicity. Recently, a study showed that proximal tubular OoC platform successfully predicted the nephrotoxicity of a drug (SPC5001). Of note, the drug exhibited nephrotoxicity in phase I clinical trials but not in preclinical animal testing on mice and non-human primates 127. A vascularized human proximal tubule model was developed in a dual-channel OoC system, which is an advancement of previous studies (Figure 3G) 128. In addition, glomerulus OoCs have been developed in the OoCs field in recent years. In a model, human glomerular endothelial cells and podocytes were seeded to reproduce the glomerular filtration barrier 35. However, other kidney structures, including the distal tubules and collecting duct, have not yet been replicated by human cells and used for toxicological applications 129.

### Gut-on-a-chip

For drug administration, the oral route is the most common. As the first step of ADME (absorption, distribution, metabolism, and excretion), absorption is the vital precondition to play the therapeutic effects of oral drugs 130. The gut is the main digestive organ, responsible for the digestion and absorption of drugs. Thus, understanding the absorption and metabolism of drugs in the gut is critical to drug discovery and development 68. The development of gut OoCs has made it feasible to study the absorption, metabolism, and transport of oral drugs. Early gut OoCs consisted of two overlapping cell culture chambers divided by a membrane lined with Caco-2 cells. To reproduce the dynamic mechanical microenvironment of the gut, this system included symbiotic microbial flora and utilized negative pressure-driven membrane stretching to simulate peristaltic movements. Under these physiological conditions, the cultured cells were reprogrammed to undergo spontaneous 3D villus morphogenesis and small intestinal cell differentiation 131, 132. Importantly, except for the barrier function of the human intestine, the model also has absorption properties that can be used for drug absorption studies 87, for example, to analyze the intestinal permeability of the model drug curcumin in real-time and generate data that are consistent with prior research on the function of the human intestinal barrier 37. Furthermore, exposure to associated biomechanical forces, like flow and peristalsis, can mimic some aspects of the drug's bioavailability and activity 79. What's more, gut OoCs are stable to create the physiologically relevant oxygen gradient support co-culture of epithelium cells with stable communities of aerobic and anaerobic gut microbiota (Figure 3F) 39, 80, which is critical for true human relevance.

### Tumor/Cancer-on-a-chip

It is still a challenge to predict clinical responses to anticancer drugs in cancer treatment 133. Tumors possess a complicated microenvironment, which contains a dense extracellular matrix (ECM), various stromal/stem and immune cells, irregular blood vessels, and limited perfusion of nutrients, all of which have a significant effect on the efficacy of administered therapies 134, 135. The advancement of cancer OoCs has significantly contributed to the capacity of *in vitro* models to reproduce the tumor microenvironment *in vivo*, as multiple factors in the tumor microenvironment (TME) can be controlled separately and precisely in microfluidic platforms, which is essential to improve anti-cancer drug selection strategies 65. A breast OoC platform mimicking cancer mammary ducts showed that tumor cells grown in channels have distinct morphologies and exhibit various sensitivities to two anticancer drugs (bleomycin and doxorubicin) compared to traditional flat surface culture 136, which provides novel insight into the development and testing of cancer therapies. Human orthotopic models of non-small-cell lung cancer OoCs can be able to simulate growth patterns observed in patients, and is consistent with the published results of human clinical trials, indicating that under physiological breathing motions, the growth and invasion of cancer cell were suppressed, and almost completely resistant to the inhibitory effects of the rociletinib 53. In addition, a pancreatic ductal adenocarcinoma OoC platform was developed to further comprehend pancreatic ductal adenocarcinoma-vascular interactions. The authors identified the activin-ALK7 pathway as a mediator of endothelial ablation by pancreatic ductal adenocarcinoma, which results in the limitation of the delivery of chemotherapeutic drugs to the tumors at later stages, and they replicated their findings in mice 137. Researchers utilized patient-specific glioblastoma OoCs to anatomize the heterogeneity of immunosuppressive tumor microenvironments and personalize anti-PD-1 immunotherapy for various glioblastoma subtypes (Figure 3E) 54. Hypoxia (Oxygen content below 3%) is a key feature of tumors, which can influence the cancer response to therapies and facilitate immune escape 138. Another bioprinted patient-specific glioblastoma OoC device reproduces clinically reported patient-specific resistances to concurrent chemoradiation and temozolomide treatment by selectively using materials with different gas-permeable properties to generate an oxygen gradient, and exhibits patient-specific sensitivity to possible drug combinations 139.

At present, tumor OoCs also include colorectal 55, 140, ovarian 141, 142, prostate 143, 144, bladder 145, 146, cervical 147, 148, gastric 56, 149, and skin 150, 151 cancer. As described, tumor OoCs have the capacity to reconstruct major tumor microenvironment characteristics and have great potential to study the mechanisms of tumor development, screen anticancer drugs, and evaluate cancer therapeutics, as well as toward precision medicine.

### Other single-organ-on-a-chip

The vasculature is important for providing adequate gas, transporting nutrients, removing waste, and offering a selective barrier for drugs introduced through the circulatory system 78, 152. As a 3D metabolically active matrix* in vitro*, which contains a capillary network for the first time that allows operating within physiological pressure gradients and interstitial flow ranges, showed an application in drug discovery 153. Meanwhile, perfusable 3D microvascular networks were successfully designed, promoting the development of vasculature OoCs 154. At present, vasculature OoCs are formed by providing endothelial cells with various chemical, cellular, or biophysical substances to induce self-assembly of the microvascular network; seeding endothelial cells onto preformed support structures (e.g., by injection molding, 3D printing, and the use of sacrificial network) or embedding cells into hydrogels, and then inducing germination by flow and chemical factors (e.g., hypoxia, VEGF, and nutrient deprivation). Recently, a vasculature OoC platform with innate immunity identified angiopoietin-1 derived peptide that can be used to therapeutic SARS-CoV-2 induced inflammation 155.

The skin acts as the largest organ in the human body and severs as the main barrier to the environment, which is critical for evaluating the cutaneous effects of drugs and modeling transdermal drug absorption 78. In recent years, drug delivery through the skin has also been a hot topic of research 112. Thus, when designing skin OoCs, the reproduction of multiple layers of skin is crucial (i.e., epidermis, dermis, and hypodermis). However, because of its complexity, it is challenging to develop a suitable substitute that can simulate all the skin's properties 156. In this context, the most common skin OoCs has been those generated by introducing directly the tissue inside the model, which continues to be regarded as the gold standard method for simulating physiological situations in a realistic setting 157, 158. Nonetheless, the variability of donor skin could affect the analysis and present challenges in evaluating compounds over time, as is typical in the drug development process. In addition, the availability is another limitation 159. Given this, both reconstructed human epidermis (including EpiDerm^TM^, EpiSkin^TM^, and SkinEthic^TM^) and full-thickness skin models have been used for many applications, such as pharmacological 160. Despite this, challenges still remain in these models to evaluate the absorption or permeability of drugs and systemic exposure with the use of topically applied drugs.

The bone is one of the active organs which is undergoing a carefully choreographed remodeling process throughout the life course 161. In recent, a microfluidic device fabricated from hydroxyapatite and PDMS provided a highly bionic bone environment. This model successfully produced a concentration gradient of the model drug, demonstrating the tremendous potential for bone-related drug screening in high-throughput 162. In addition, a vascularized human bone marrow OoC platform containing bone marrow-derived stromal cells and CD34^+^ cells, which can generalize myeloerythroid toxicity following exposure to chemotherapeutic agents 45. Another bone marrow OoC device consists of the human endosteal, central marrow, and perivascular niches, which can be utilized to obtain a better understanding of normal and impaired hematopoiesis, and a variety of bone marrow pathologies (Figure 3C) 46.

Human donor retinal explants offer a fully functional model, but due to inter-donor variability, limited availability, and poor cultivability, it is not suitable for drug development and testing 47. Recently, a study demonstrated that the interaction of mature photoreceptor segments with the retinal pigment epithelium (RPE) can be reproduced *in vitro* by a retina OoC platform, which was integrated with over seven different hiPSC-derived essential retinal cell types. Importantly, the model recapitulated the retinopathic side effects of the antibiotic gentamicin and the anti-malaria drug chloroquine, exhibiting the potential of facilitating drug development 47. As another example, a model supporting the outer blood-retinal barrier (oBRB) barrier function successfully mimicked the pathogenesis of choroidal neovascularization (CNV, a key pathological step in a variety of ophthalmic diseases), and proved that bevacizumab alleviated pathological angiogenesis (Figure 3D) 48.

Nowadays, muscle OoCs have been employed in mechanistic research to better comprehend the human skeletal muscles and assess the effects and toxicity of drugs 163. The development of safer and more effective drugs could be helped with the obtained accurate contractility data. Given this, a muscle thin-film technology-based muscle OoC platform demonstrated its ability to simultaneously analyze the contractility of both striated and smooth muscle on the same chip 164. Recently, a high-throughput aorta smooth muscle OoC device replicated the abnormal activation of HIF-1α observed in aortas from thoracic aortic aneurysm patients, and finally identified the two most effective drugs (2-methoxyestradiol and digoxin) from the seven specific HIF-1α inhibitors 49.

Fat tissue, as a major energy reserve, will contribute to obesity due to the imbalance between energy intake and expenditure, and its associated comorbidities present a looming challenge to healthcare delivery throughout the world 165, 166. The interaction of immune cells and adipocytes may lead to chronic low-grade inflammation, which will then result in insulin resistance. An OoC system for characterizing the interaction of adipocytes with immune cells displayed increased pro-inflammatory cytokine secretion and insulin resistance, relative to adipocytes alone. Compared to the previously reported data, the known diabetic drug metformin and the nutraceutical compound omega-3 showed satisfactory results (Figure 3H) 51. Another OoC platform allows monitoring of the intake of fatty acids and quantification of metabolite released into the effluent media in real-time, and its applicability for pharmaceutical research has been assessed by using isoproterenol, which is known to induce lipolysis 52.

## The application of multi-organ-on-a-chip in drug evaluation

**Figure 4:**
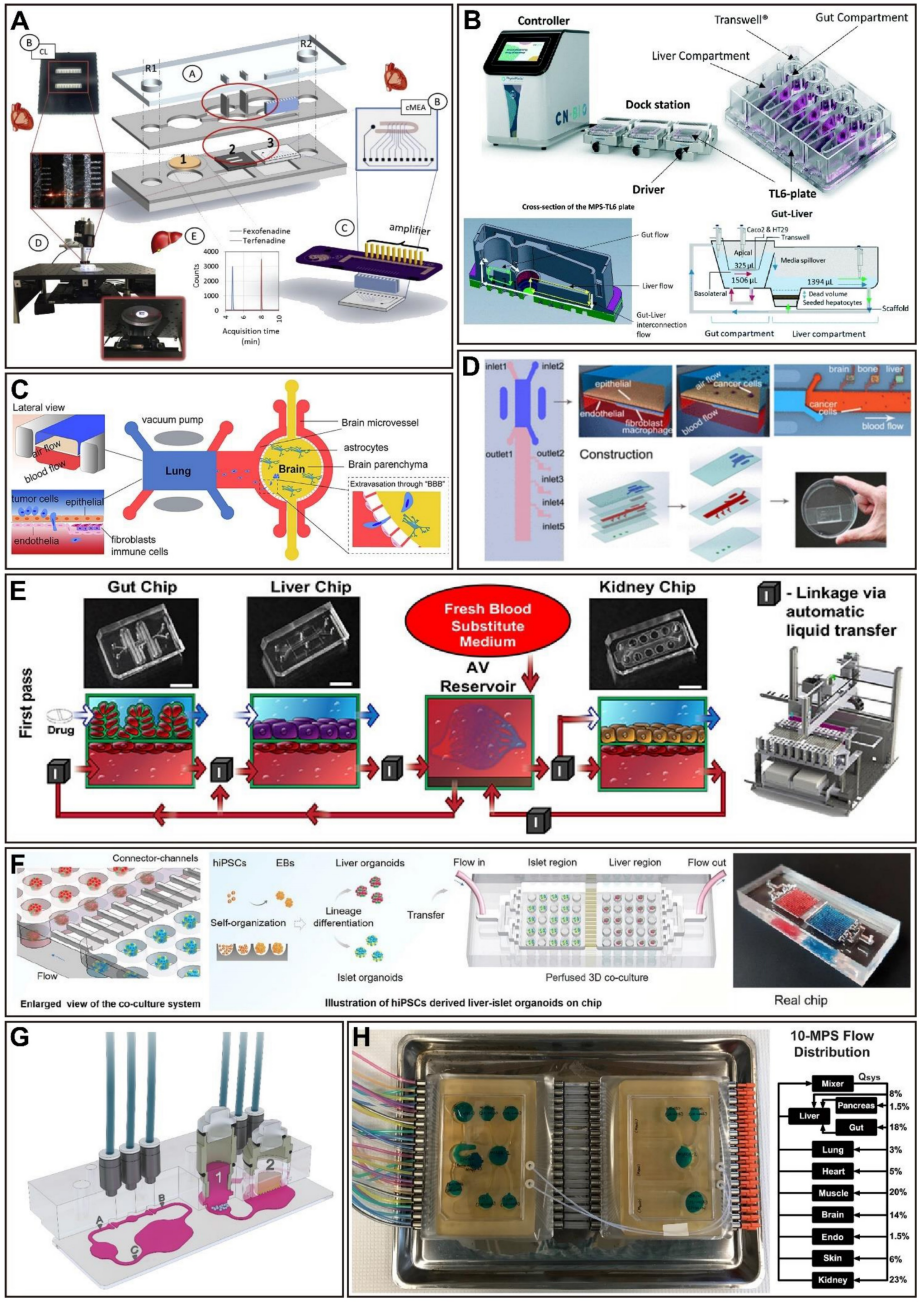
** Representative examples of multi-OoCs. (A)** A Liver-heart platform for studying the effect of liver metabolism on off-target cardiotoxicity. Adapted with permission from 170, copyright 2018 Elsevier. **(B)** PhysioMimix gut-liver MPS consists of a controller machine with a pump system and a touchscreen that interacts with the user. The system was used for the quantitative pharmacokinetic study of mycophenolate mofetil. Adapted with permission from 180, copyright 2022 Royal Society of Chemistry. **(C)** A multi-OoC platform consisted of two bionic organ modules, an upstream 'lung' and a downstream 'brain', allowing to study of lung cancer brain metastasis. Adapted with permission from 187, copyright 2019 Elsevier. **(D)** A four-organ system for mimicking lung cancer cell metastasis to the liver, bone, and brain. Adapted with permission from 188, copyright 2016 American Chemical Society. **(E)** A multiple vascularized OoC platform utilizing fluid transfer coupling enables quantitative prediction of human PK responses. Adapted with permission from 177, copyright 2020 Springer Nature. **(F)** The differentiation and generation of hiPSCs-derived liver and islet organoids in a microfluidic device to simulate human-relevant liver-islet axis under both physiological and pathological conditions for future T2DM study and drug development. Adapted with permission from 183, copyright 2022 Wiley. **(G)** A 3D co-culture microfluidic model for simultaneous assessment of anti-EGFR-induced tumor and adverse skin impacts. Adapted with permission from 198, copyright 2018 Nature Publishing Group. **(H)** A multi-OoC system containing up to 10 different organs with different flow configurations, which include epithelial barrier tissues and non-barrier organs, for PK analysis of diclofenac metabolism. Adapted with permission from 182, copyright 2018 Nature Publishing Group.

As the development of single OoCs matures, when these single organs are fully functionally characterized (i.e., when they show the key characteristics of the desired simulated organ), they can be combined to create the proposed multi-organ-on-a-chip (often referred to as body-on-a-chip or human-on-a-chip). Connecting a single OoCs to another by microfluidics simulates the *in vivo* role of vascular perfusion and allows control of the culture environment to reproduce some aspects of homeostasis 78. The main advantage of multi-OoCs is obvious, that is, these connections enable the complex and dynamic crosstalk between interested organs and promote a more physiological method for drug delivery, distribution, and absorption 167. As reported, there are three main strategies for connecting single OoCs: 1) connecting the single organ modules with the use of capillary tubing; 2) attaching single organ modules to a microfluidic motherboard that contains all fluidic connections; 3) employing a user-friendly plate with all organ models connected to a channel that controls fluid flow in a manner similar to the vasculature 89. So far, multi-OoCs may have from 2 to 10 different organs, generally between 2 to 4 organs, which have been capable of simulating complex physiological and pathophysiological responses in an impressive manner, and also offer new *in vitro* tools for assessing drug toxicity and PK/PD 73, finally towards personalized medicine. In this section, we focus on reviewing the main application scenarios of multi-OoCs (Figure 4 and Table 2).

### Drug safety evaluation

In most cases, many drugs fail in phase III clinical trials or have serious side effects after marketing 78, leading to failure in the development of new medicine. Thus, the evaluation of toxicity is critical in late-stage preclinical and clinical research. Toxicity is closely related to liver metabolism, so multi-OoCs designed for toxicity purposes typically include a liver (as the primary site of drug metabolism) and at least one other (target) organ. For example, a biomimetic human liver OoC platform with lobule-like microarchitectures successfully analyzed unfavorable reactions caused by drug-drug interactions of clinical pharmaceuticals during hepatic metabolism, providing an evaluation device to assess drug-induced hepatotoxicity *in vitro*, especially during combinational therapies 168. In addition, the use of the lung-liver OoC system in acute and chronic toxicity studies of drugs provides new opportunities for demonstrating the security and effectiveness of new drug candidates that target the lung 169. A model with primary hepatocytes and iPSC-CMs allows non-invasive readouts of the cardiotoxicity of drugs and their metabolites while also exploring the impact of liver metabolism on off-target cardiotoxicity, which demonstrates the heart-liver crosstalk (Figure 4A) 170. In a subsequent study, a heart-liver platform containing a skin mimic showed the differential effects of acute and chronic drug exposure, which can be utilized to assess potential drug toxicity from dermal absorption 171. Moreover, multi-organ toxicity was exhibited in a four-organ system made up of neuronal, muscle, cardiac, and liver modules, and all drug treatments generally agreed with published toxicity results based on human and animal data 172. Additionally, a system that integrates liver, lung, cardiac, colon, testis, vascular, and brain derived from human primary cells and stem cells, which can stay viable for at least 28 days, responding appropriately to a series of drugs, including those because of toxicity in humans that the FDA has removed from the market 173. The promise of OoCs to promote drug development lies in their ability to provide humanized drug toxicity information, which can be used as a useful tool to assess drug toxicity effectively and accurately prior to the drug being approved for use in clinical trials.

### Drug PK/PD modeling

After identifying candidate molecules and targets, PK and PD studies are conducted. On the one hand, PK researches describe drug concentrations at various organ sites during metabolism, which is referred to as the absorption, distribution, metabolism, and elimination (ADME) of drug candidates. On the other hand, PD researches investigate the effects of the drug on target organs or tissues, such as a correlation between drug dose and pharmacological or toxicological response 174. The combination of PK/PD parameters is critical for new drug development because it can predict the drug response that will occur, thus minimizing the production of toxic metabolites and the side effects of drugs 175, 176. For instance, a multi-OoC platform allowed recapitulation of physiological PK modeling of drug absorption, metabolism, and excretion which drugs first-pass. The model was verified using orally administered nicotine (using gut, liver, and kidney chips) and intravenously injected cisplatin (using coupled bone marrow, liver, and kidney chips). Also, the cisplatin PD predictions are consistent with previously published patient data 177. Determination of drug-administration schemes for phase I clinical trials may be improved by the quantitative *in-vitro*-to-*in-vivo* translation of PK and PD parameters via fluidically coupled OoCs (Figure 4E) 177. In a recent study, a platform that adopted a multi-layer structure was used to systematic analysis the absorption, metabolism, and toxicity of ginsenoside compound K, and the PK results were consistent with previous reports 178. Another multi-OoC platform called 'HUMIMIC Chip2' was used to integrate liver spheroids and a skin model for PK-PD studies with local exposure to chemicals of hyperforin and permethrin 179. Moreover, integrating gut-liver OoC platform data with *in silico* modeling allows to investigate complex combinations of intestinal and hepatic processes for quantitative *in vitro* PK studies (Figure 4B) 180. Recently, OoCs that combine more organs are designed to study PK/PD. A robotic interrogator maintained the viability and organ-specific functions of eight vascularized, two-channel OoC devices (liver, heart, kidney, intestine, skin, lung, brain, and BBB) for 3 weeks in culture, and predicted the distribution of an inulin tracer throughout the entire system 181. Furthermore, a three-layer microbioreactor-based platform containing up to ten different organs, including epithelial barrier tissues and non-barrier organs, which can sustain cell cultures for more than four weeks. The functionality of the platform has been verified by modeling the PK of a nonsteroidal anti-inflammatory drug diclofenac, which revealed that both diclofenac and 4-OH-diclofenac were distributed throughout all the representative organs (Figure 4H) 182. In summary, the outcomes from multi-OoCs provided insightful data that eventually be applied to evaluate the PK/PD of potential new drugs, leading to a more dependable preclinical stage in drug development.

### Disease modeling

The dearth of clinically applicable models is a challenge for many human diseases, especially those complex diseases that involve multiorgan interactions. As a systematic multi-organ metabolic disease, Type 2 diabetes mellitus (T2DM) is characterized by the dynamic interplay of various organs 183, and a clinical cure is not yet available. Recently, a multi-OoC platform was used to model the liver-pancreatic islet axis under both normal and type 2 diabetes conditions, which successfully mimicked the functional coupling of the liver and islet organs' response to external hyperglycemic stimulus and drugs is relevant to humans (Figure 4F) 183. An ulcerative colitis multi-organ system was created by connecting the liver, gut, and circulating immune cells, showing that short-chain fatty acids (SCFAs) derived from the microbiome could either improve or worsen the severity of ulcerative colitis, and these converse results resting with the participation of effector CD4^+^ T cells 184. This study brought new insights into the immune and metabolic regulation of pathophysiology. Moreover, multi-OoCs connected to the vasculature and circulatory system are critical for understanding local and distant disease development, like cancer initiation and metastasis 185, the latter contributes to up to 90% of cancer-related mortality 186. For instance, a methodological platform that was used to study brain metastasis demonstrated that the protein Aldo-keto reductase family 1 B10 (AKR1B10) contributes to brain metastasis of lung cancer cells (Figure 4C) 187. Furthermore, a four-organ platform that reproduced lung cancer metastasis to the liver, bone, and brain, revealed tumor-induced tissue damage in the targeted bone and liver compartments (Figure 4D) 188. These suggest that multi-OoCs are a practical alternative for predicting cancer metastasis and evaluating antimeatstatic therapies. In addition, the advancement of degenerative brain diseases including Parkinson's or Alzheimer's disease has been linked with gut microbiota, this functional relation is often referred to as the microbiota-gut-brain axis (MGBA) 189. However, a comprehensive *in vitro* model was lacking for researchers to elucidate potential microbiota-neurodegeneration mechanisms. The European Research Council has funded a project called 'MINERVA' (ID 724734), which seeks to build the first multi-OoC device for microbiome-gut-brain engineering to assess the effect of intestinal microflora on neurodegeneration 190. More importantly, multi-OoCs are particularly valuable for clarifying mechanisms and developing treatments for rare diseases affecting multiple organ systems, where drug development is incredibly difficult because of the available human subjects being scarce, like Churg-Strauss syndrome and POEMS syndrome 191. Thus, the application of multi-OoCs to model diseases improves disease comprehension, diagnosis, prevention, and treatment.

### Personalized medicine

Although various* in vitro* platforms have been developed for drug development screening, there are few that exist for clinical deployment to benefit unique patients. This is an unmet clinical need because patient responses to drugs are frequently unpredictable due to genetic and microenvironmental heterogeneities 192. Remarkable strides in the hiPSCs field allow for the development of patient-specific personalized therapies, making it possible to identify more efficient drugs for a particular individual or patient group 175. Recently, the integration of a heart chip and a liver chip, both created with the same hiPSC line, was reported to investigate the drug-drug interaction (DDI) of the fungicide ketoconazole and the arrhythmogenic gastroprokinetic cisapride, which facilitates the screening of DDI 193. In the treatment of COVID-19, more attention should be paid to comorbidities. A lung OoC platform comprising infected cells from COVID-19 patients has the promise to overcome the potential effects, such as liver, cardiovascular, and kidney disease, or malignant tumors, as which have occurred in patients reported previously, and may assist in providing effective treatment for individual patients 175, 194. Oncology diseases, which are characterized by rapid mutations that lead to morphological changes and various phenotypes of multidrug-resistant that affect the patient's response to treatment, are another area where multi-OoCs have gained great attention in personalized treatment 195, 196. A multisensor-integrated multi-OoC system was developed, and by linking iPSC-CMs and primary hepatocytes together to achieve automated sensing of APAP-induced organoid toxicity. Using this model, hepatocytes were replaced by hepatocarcinoma cells to assess the chemotherapeutic drug doxorubicin treatment-induced pronounced cardiotoxicity 197. Thus, this platform can be used in predicting the cardiotoxicity of drugs by using patient-specific iPSC-CMs. Moreover, a commercially available multi-OoC platform coupling two organs (lung cancer and skin) culture compartments fluidically for evaluating the efficacy of therapeutic anti-EGFR monoclonal antibodies while analyzing a side effect of dermatological toxicities. The results showed that it is possible to detect several key side effects on the cetuximab-exposed skin microtissues at a very early stage, as well as reproduce the inhibition of keratinocyte growth and altered expression of CXCL8 and CXCL10 observed in patients 198. We believe it will be achievable to personalize the screening of drugs using patient-specific preclinical models prior to treatment, while monitoring the adverse effects of all organ systems in the platform, and improving treatment outcomes. Of note, a four organ model integrated predifferentiated organs from the same human iPSCs and successful coculture over 14 days (although the renal model did not further differentiate) 199, which demonstrates the promise of taking advantage of OoCs to optimize the selection of therapeutics in a personalized manner.

## Integration OoC technology with artificial intelligence

OoC technology and deep learning are frontier fields in biomedical engineering and AI, respectively, and represent an ideal combination of experimental and analytical throughput 60. Here, we introduce various applications of AI to OoCs, trying to illustrate the power and versatility of integrating OoCs with AI. Although the integration of these two disciplines has not been extensively explored so far, especially in the field of drug evaluation, we can still get a glimpse of the great potential of OoCs combined with AI in future drug evaluation from the existing research.

### The challenges faced by OoCs in higher-throughput

**Figure 5:**
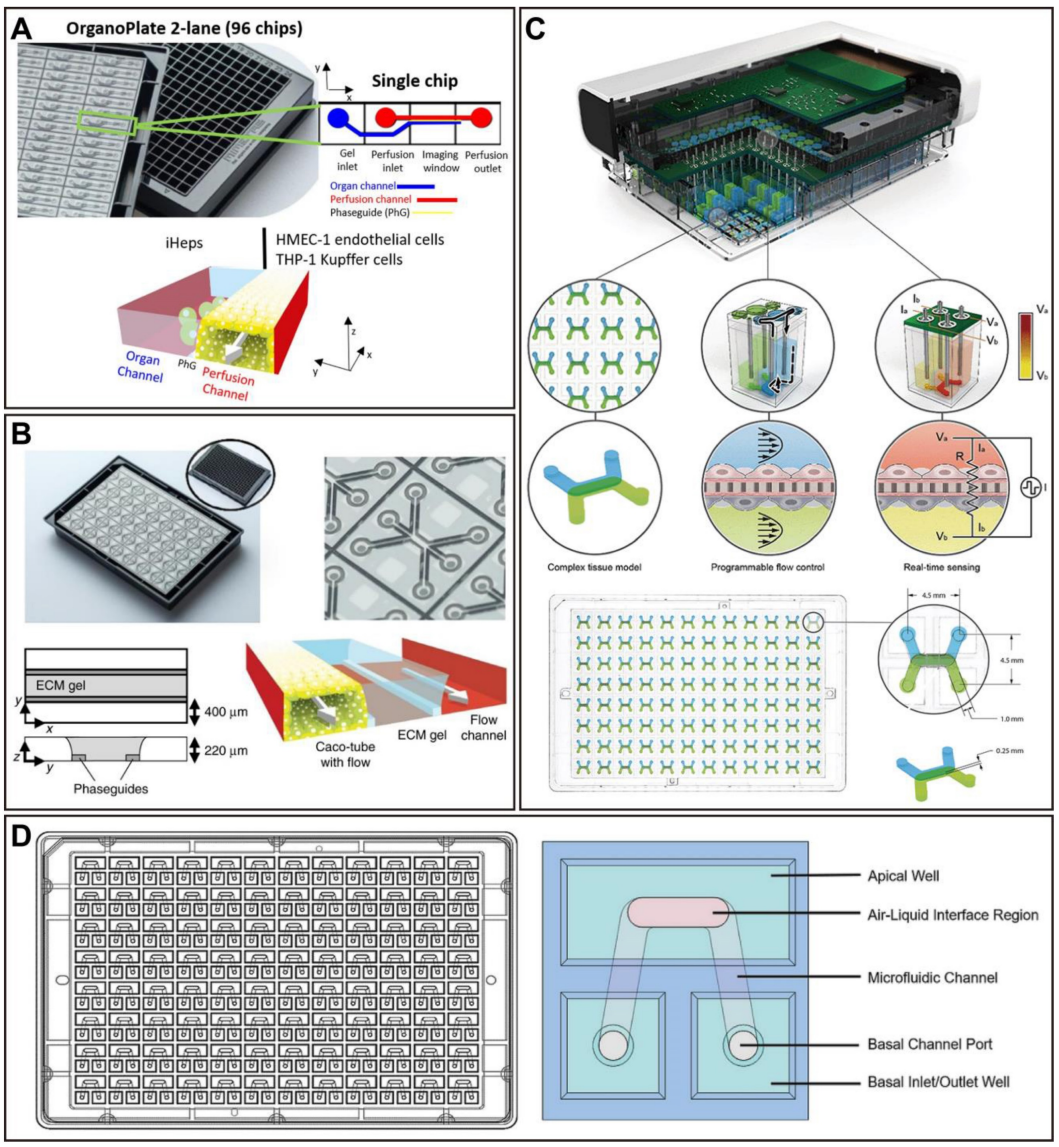
** Representative examples of high-throughput implemented in OoCs. (A)** An OrganoPlate 2-lane has 96 chip units, with the perfusion and organ channels divided by a Phaseguide. Adapted with permission from 31, copyright 2021 Elsevier. **(B)** An OrganoPlate contains 40 microfluidic channel networks, each of which consists of three channels (including a tubule with flow, an extracellular matrix gel, and a flow channel) that join in the center. Adapted with permission from 204, copyright 2017 Nature Publishing Group. **(C)** A platform incorporates 96 independent microfluidics-based organ models, each with two channels separated by a permeable membrane, and the micropumps integrated with the trans-epithelial electrical resistance electrodes and electronics of the micro-pump sensor array. Adapted with permission from 202, copyright 2021 Royal Society of Chemistry. **(D)** A PREDICT96-ALI platform is a standard 384-well plate layout, which is an individual airway model with an oval-shaped upper chamber and an inverse U-shaped bottom chamber with inlet and outlet ports. Adapted with permission from 203, copyright 2021 Nature Publishing Group.

High-throughput platforms for preclinical drug screening are crucial to reducing the cost of drug discovery 200. Nowadays, the relatively low throughput of the majority of OoC platforms has hampered the widespread adoption of organ-on-a-chip for drug screening. More reliable statistical data requires a large number of tests and results, hence the need for higher-throughput studies on OoCs. Recently, a few studies have been proposed to address this need (Figure 5). For instance, a microfluidic device for modeling the human microcirculation was demonstrated, as a protocol extension. This device can self-organize human microvascular networks, and then perfuse the tumor to summarize discrete steps of early metastatic seeding. Combined with high-resolution imaging, reliable and quick scoring of extravascular cells can be easily achieved. In addition, the ability to manufacture and seed up to 36 devices at once while not affecting cell viability was reported, which further allows for highly parametric studies, and generating a significant amount of data 201. A high-throughput OoC platform with 96 devices integrated programmable fluid flow and real-time sensing for physiologically relevant tissue generation and measurement, enabling accelerated optimization of *in vitro* models (Figure 5C) 202. Another 96-device platform (PREDICT96-ALI) is compatible with high-resolution *in situ* imaging and real-time sensing for rapid assessment of drug efficacy against viruses including coronaviruses (Figure 5D) 203. In perfused microfluidic devices, extracellular matrix-supported intestinal tubules were introduced. The OrganoPlate platform is a standard 384-well microtiter plate format with 40 microfluidic channel structures. On this platform, a study containing 357 gut tubes was conducted to test against drug compounds at various concentrations to evaluate the impact on epithelial barrier integrity. Notably, the study produced more than 20,000 data points, which makes it the largest reported OoC platform data set to date (Figure 5B) 204. Another microfluidic platform named IFlowPlate was also built on a 384-well plate, which can be used to culture up to 128 organoids, achieved *in vitro* perfusable culture and vascularization of patient-derived colon organoids, and successfully developed a colon inflammation model with an innate immune function 205. Thus, the higher-throughput nature of these studies suggests the potential of OoCs as novel, effective, and dependable preclinical models with applications in drug evaluation.

However, it must be recognized that growing throughput typically causes large data generation, leading to labor-intensive and time-consuming processes. Thus, in order to streamline the experimental procedures, it is crucial to develop protocols that facilitate efficient device operation, data collection, and data analysis. For instance, robotics can be used to automate tasks (e.g., operating chips and gathering data), while machine learning can be used to speed up data analysis 93.

### The increasingly prominent advantages of AI

In the past few years, AI has supplied significant advantages in many areas of healthcare in research and clinical settings, such as disease diagnosis, precision medicine, and drug discovery and development. Notably, opportunities for applying AI arise at all stages of drug discovery and development, including clinical trials 206. Applications include identification and validation of drug targets, designing of new drugs, quantifying structure-activity relationship, drug repurposing, improving the research and development (R&D) efficiency, as well as evaluation of absorption, distribution, metabolism, excretion, and toxicity, and even aggregating and analyzing biomedicine information and refining the decision-making process to recruit patients for clinical trials and so on 207-210. Furthermore, the identification of new disease genes, pathways, and targets using omics analysis with AI becomes possible 211, 212, thereby providing new mechanisms for future drug discovery and development, as well as precision medicine. Facing massive volumes of accumulated data (e.g., medical images and gene expression data), AI-based approaches can further transform these enormous amounts of data into usable knowledge, thus facilitating systematical discovery, understanding, and learning 213. Importantly, the application of AI offers the opportunity to overcome the inefficiencies and uncertainties in traditional drug discovery and development approaches, while also reducing human intervention and personal bias in the process 208. Today, advances in areas OoCs and AI are increasingly providing the basis for more efficient and successful drug evaluation. Notably, multi-OoCs, and future coupling with AI, will provide a powerful tool for the pharmacological research of drugs, especially complex chemical drugs, botanical medicines, and Traditional Chinese Medicines.

ML is a common technical means to achieve AI, and DL is a type of ML algorithm. Of note, DL is the most representative research field in AI 19. ML could be categorized into supervised, unsupervised, semi-supervised, and reinforcement learning based on labels 214. ML provides automated analytical statistical/model-building approaches for machines to make decisions by extracting information from data or identifying patterns (i.e., learning), without explicit human programming 215. ML has been growing utilized to analyze data (e.g., to clarify processes and predict outcomes), which may reduce inter-operator variability during data analysis. Deep learning is a machine learning technique encompassing a variety of learning models known as deep neural networks (DNNs), which are referred to as 'deep' since containing multiple processing layers 213, 216. The blooming of algorithms, including Deep Belief Networks (DBNs), Autoencoder Networks (AEs), Convolutional Neural Networks (CNNs), Recurrent Neural Networks (RNNs), and Generative Adversarial Networks (GANs) 217, 218, leading to various studies with the use of DL-based AI in drug evaluation. In comparison to traditional ML, which has a limited ability in processing natural data in its raw form, while DL directly performs the feature extraction of the data 216, and is easier to have high accuracy by minimizing errors. Moreover, DL models previously trained on one task commonly could be retrained to execute similar tasks, named transfer learning, which typically needs raw data and fewer computational resources, making DL applicable to a variety of tasks 219.

With the development of OoCs, especially utilizing higher-throughput, highly parallelized microfluidic systems, generating unprecedented quantities of data; however, the large amount of data generated has far exceeded researchers' capacity to process it efficiently, creating a bottleneck in the analysis 60. Typically, manually analyzing data is inefficient and is likely to miss trends that are nonobvious or of interest 220, hence the need for appropriate systems to manage and analyze the data. Thus, AI has been applied to address the challenge of analyzing large and multidimensional datasets, and assists researchers to derive meaningful insights. In brief, new data processing systems should contain four main components: 1) the suitable measuring hardware and microchips, with precise sensors and microsystems to effectively monitor the required parameters; 2) the provided appropriate forms of data collection, transmission, and storage; 3) the improved machine learning algorithms which enable to extract desired information from the obtained massive amounts of data sets; 4) the proper explanation of the collected data, and applied to the discovery of new results 65, 213.

### AI-based visual recognition in data analysis of OoCs

To date, the most common data output structure for OoCs is fluorescence microscopic images 60, due to the PDMS's transparency and great compatibility with fluorescence microscopy. This is traditionally handled by manual methods, which are frequently inefficient, time-consuming, and error-prone 221. However, it is important to note that DNN has been trained with organ autofluorescence images to achieve virtual histological staining for relevant assays, including hematoxylin-eosin (H&E), Masson's trichrome, and Jones silver stain. The outcomes showed that these computational stains are almost indistinguishable from the corresponding histologically stained tissue 222. Thus, virtual staining of label-free samples on OoCs could be achieved by utilizing deep learning in the near future.

**Figure 6:**
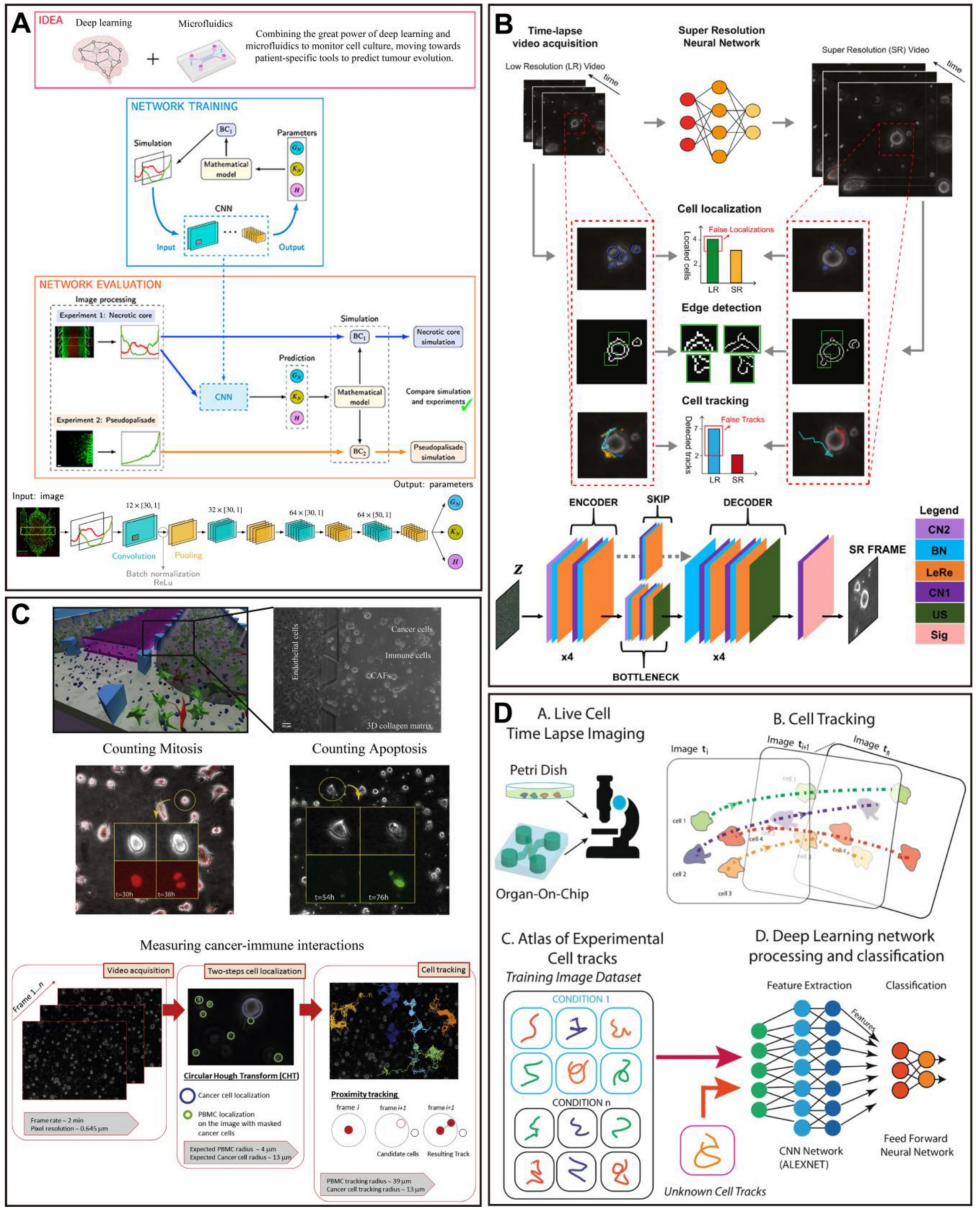
** Representative examples of OoCs integrated with AI for data analysis. (A)** A CNN model was developed to identify important tumor cell behavior parameters from fluorescence images in a glioblastoma OoC platform, and combined with an* in vitro*-*in silico* approach to achieve real-time prediction of tumor evolution. Adapted with permission from 223, copyright 2021 Elsevier. **(B)** A deep prior algorithm, called Recursive Deep Prior Video, was developed for addressing the challenge of the low resolution in time-lapse microscope in OoC applications, and the approach was successfully validated. Adapted with permission from 225, copyright 2021 Elsevier. **(C)** A tumor OoC platform combining cancer, immune, endothelial, and fibroblasts recapitulated an anti-tumoral antibody-dependent cell-mediated cytotoxicity, and CellHunter method was used to track cancer-immune cell interactions. Adapted with permission from 233, copyright 2018 Cell Press. **(D)** The cells on the OoC are located and tracked through the video sequence obtained by time-lapse microscopy, and then extract relevant features from the visual atlas are for classification tasks with the use of a pre-trained DL-based algorithm. Adapted with permission from 228, copyright 2020 Nature Publishing Group.

**Figure 7:**
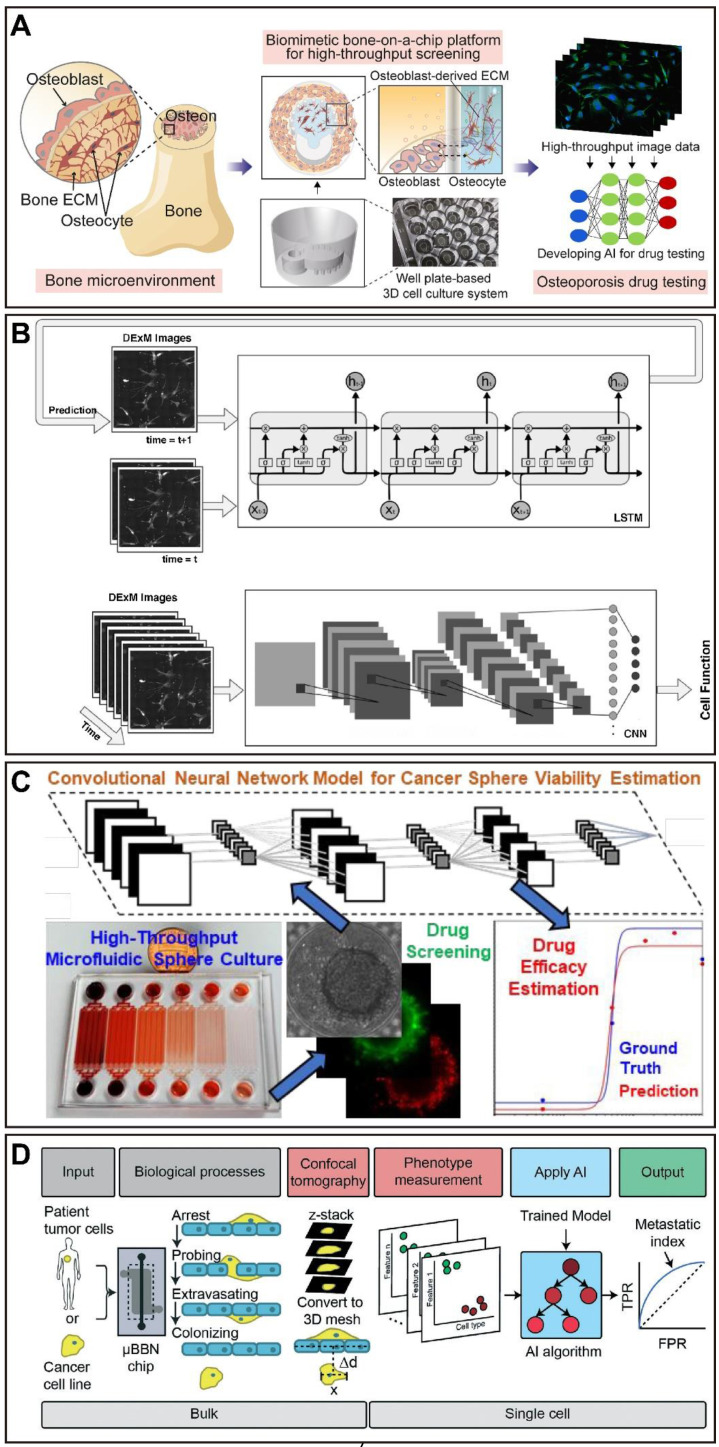
** Representative examples of OoCs integrated with AI for data analysis. (A)** A biomimetic bone OoC device for high-throughput osteoporosis drug testing with AI-assisted image analysis. Adapted with permission from 226, copyright 2022 Wiley. **(B)** In a muscle OoC device, temporal prediction of muscle cell morphology was studied with the RNN model with long short-term memory blocks and trained a CNN model with the temporal images of the RNN to judge the cell function. Adapted with permission from 227, copyright 2019 Elsevier. **(C)** A microfluidic platform with up to 1920 tumor spheres integrated with a CNN model to assess the efficacy of chemotherapy drugs. Adapted with permission from 230, copyright 2019 American Chemical Society. **(D)** A µBBN platform for identifying the extravasation potential of cancer cells to brain metastasis niches with advanced live cell imaging algorithm and artificial intelligence. Adapted with permission from 232, copyright 2019 Royal Society of Chemistry.

**Figure 8:**
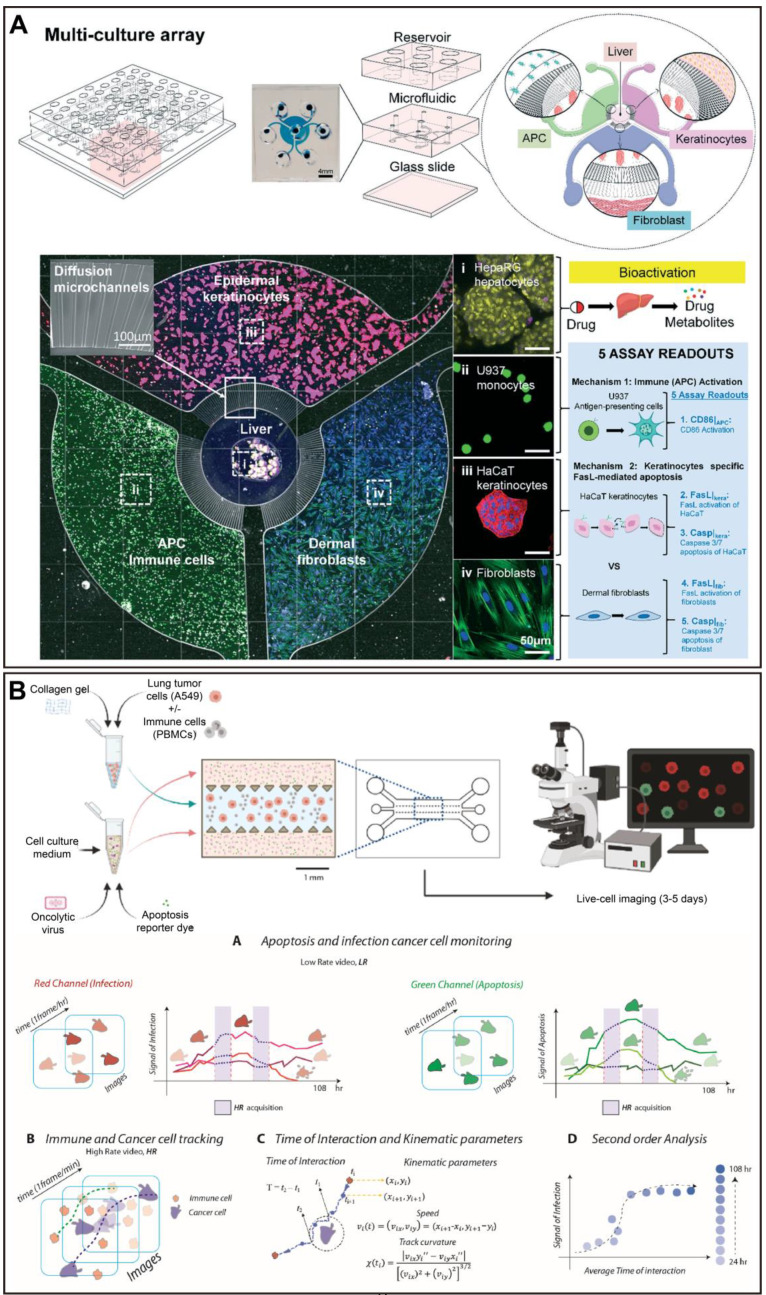
** Representative examples of OoCs integrated with AI for data analysis. (A)** A microfluidic multicellular coculture array (MCA) was developed and combined with ML to assess adverse skin drug responses. Adapted with permission from 224, copyright 2022 Royal Society of Chemistry. **(B)** The combined anti-tumor activity of immune cells and oncolytic vaccinia virus in tumor OoC device was revealed by direct imaging and automated analysis. Adapted with permission from 237, copyright 2022 Elsevier.

In a recent study, a CNN model was developed to identify important tumor cell behavior parameters from fluorescence images in a glioblastoma OoC platform, and combined with an *in vitro*-*in silico* approach to achieve real-time prediction of tumor evolution (Figure 6A) 223. A microfluidic multicellular coculture array (MCA) was developed and combined with ML to assess skin sensitivity to drugs. The performance prediction of MCA and support vector machine (SVM) classification algorithm demonstrated that the model has 87.5% accuracy, 75% specificity, and 100% sensitivity in predicting skin adverse drug reactions, with the potential as a platform for high-throughput drug screening (Figure 8A) 224. For some OoC platforms, the segment of the special parts of the image with great significance is important for the analysis of experimental results. In this case, applying DL models to accomplish pixel-level segmentation of images acquired from OoCs, will help in the analysis of drug therapy 19. Given this, researchers have developed a new DL-based algorithm without requiring any training, called Recursive Deep Prior Video, to address the challenge of the low resolution in time-lapse microscope in OoC applications, and the approach was successfully validated on real videos of OoC experiments associated with tumor-immune interactions (Figure 6B) 225. Furthermore, a biomimetic bone OoC device has been developed to achieve simultaneous and high-throughput drug testing for osteoporosis. This device was integrated with CNN-based image segmentation algorithms for fluorescence image analysis, and successfully validated its feasibility for drug evaluation (Figure 7A) 226, although mouse-derived cells were used, human-derived cells can be used for future studies.

At the same time, the possibility to visualize cell morphology and trajectory in real-time is essential for the study of drug therapy in OoCs, especially in cancer OoCs. For example, in a muscle OoC device, the authors studied the temporal prediction of muscle cell morphology with the use of an RNN model with long short-term memory blocks. Next, a CNN model was trained with the temporal images of the RNN to judge the cell function (Figure 7B) 227. Besides, a cancer OoC system with the use of a DL-based CNN architecture was used to evaluate the effectiveness of cancer drug therapies by discovering the hidden information within cell trajectories. The Deep Tracking was capable of accurately classifying the cells (91.5% on average), indicating that the DL approach is very proficient at identifying how drug treatments affect cell motility behaviors (Figure 6D) 228. Notably, the use of a CNN algorithm for recognition achieved to perform accurate tumor boundary detection and analysis of tumor invasion 229, which could be further applied in OoCs. Another platform supported by automated image acquisition and cropping analysis has successfully implemented a label-free approach to evaluate the viability of tumor spheroids on a microfluidic platform with up to 1920 tumor spheroids. The authors trained a CNN model to estimate sphere viability based on bright-field images, and accurately evaluated the efficacy of three chemotherapeutic drugs, adriamycin, oxaliplatin, and irinotecan. It is important to note that the training networks of doxorubicin and oxaliplatin have been cross validated, indicating the possibility of using representative drugs to train a universal network and applying it to numerous different drugs in large-scale screening (Figure 7C) 230.

A key challenge in cancer studies is the increased complexity of the tumor microenvironment. Recently, a novel 3D microfluidic blood brain niche (µBBN) platform quantified the microenvironment of brain metastatic tumors (breast cancer) by using confocal tomography and machine learning (neural networks and random forest learning algorithms) to identify intrinsic phenotypic differences in tumor cells capable of metastasizing through the model 231. The same group performed a similar study prior to this one, in which breast cancer cell extravasation was analyzed within a μBBN device using an advanced live cell imaging algorithm to detect small differences between cells with and without brain metastasis potential (Figure 7D) 232. The device enables further utilization to assess the molecular determinants of metastatic cancer cell migration and survival, and to evaluate the effectiveness of drug therapy. In addition, a more complex and better replicating breast tumor microenvironment was built and CellHunter was employed to track intercellular interactions between immune cells and tumor cells in OoCs (Figure 6C). Deep learning enables visualization and quantification of the complex dynamics of tumor OoCs, for characterizing drug responses at the ecosystem level, and for dissecting the roles of stromal components 233.

CellHunter is a DL-based cell tracking analysis algorithm. As reported, CellHunter has been successfully applied to reveal the interactions between human peripheral blood mononuclear cells (PBMCs) and tumor cells in OoCs and showed that only cells from 'wild type' donors (FPR1 normal expression) establish sustained interactions with chemotherapy-treated cancer cells 234. Another similar study investigated the effect of spatial and temporal resolutions of cell-cell interaction analysis in OoCs based on the same platform 235. Furthermore, a microfluidic platform combining advanced microscopy and revised CellHunter was developed to assess the effective migration of interferon-α-conditioned dendritic cells (IFN-DCs) toward drug-treated cancer cells, while discovering the involvement of major underlying factors, such as CXCR4 236. The oncolytic vaccinia virus is an emerging agent in cancer immunotherapy. Recently, a tumor OoC device revealed cooperative antitumoral activity of immune cells and oncolytic vaccinia virus by direct imaging and automatic analysis. In this work, the CellHunter algorithm was applied to high-resolution video analysis to localize and track cancer cells, which high-resolution video is aimed at measuring immune kinematics and cancer-immune interactions (Figure 8B) 237.

Angiogenesis has been reported to be associated with more than 70 diseases. Lately, a study has utilized DL-based image processing algorithms to analyze and quantify angiogenesis on a chip. This method allows the assessment of angiogenesis using up to 16 angiogenesis-related metrics and the extraction of 3D indicators from 2D images. In this work, the model successfully showed changes in response to biochemical gradients and can be applied to drug development 238. Moreover, a new OoC platform for growing, vascularized, and perfused microtissues suitable for large-scale drug screening has been applied to assess the effectiveness of antiangiogenic compounds. This platform is based on a CNN method that enables fast and accurate flagging of compounds that effectively disrupt vascular networks from images of before and following drug applications with near-perfect accuracy. The CNN model significantly outperformed well-trained human raters, representing a substantive step in the automated analysis of data toward high-throughput drug screening 239.

### AI-based in electrochemical detection and analysis of OoCs

**Figure 9:**
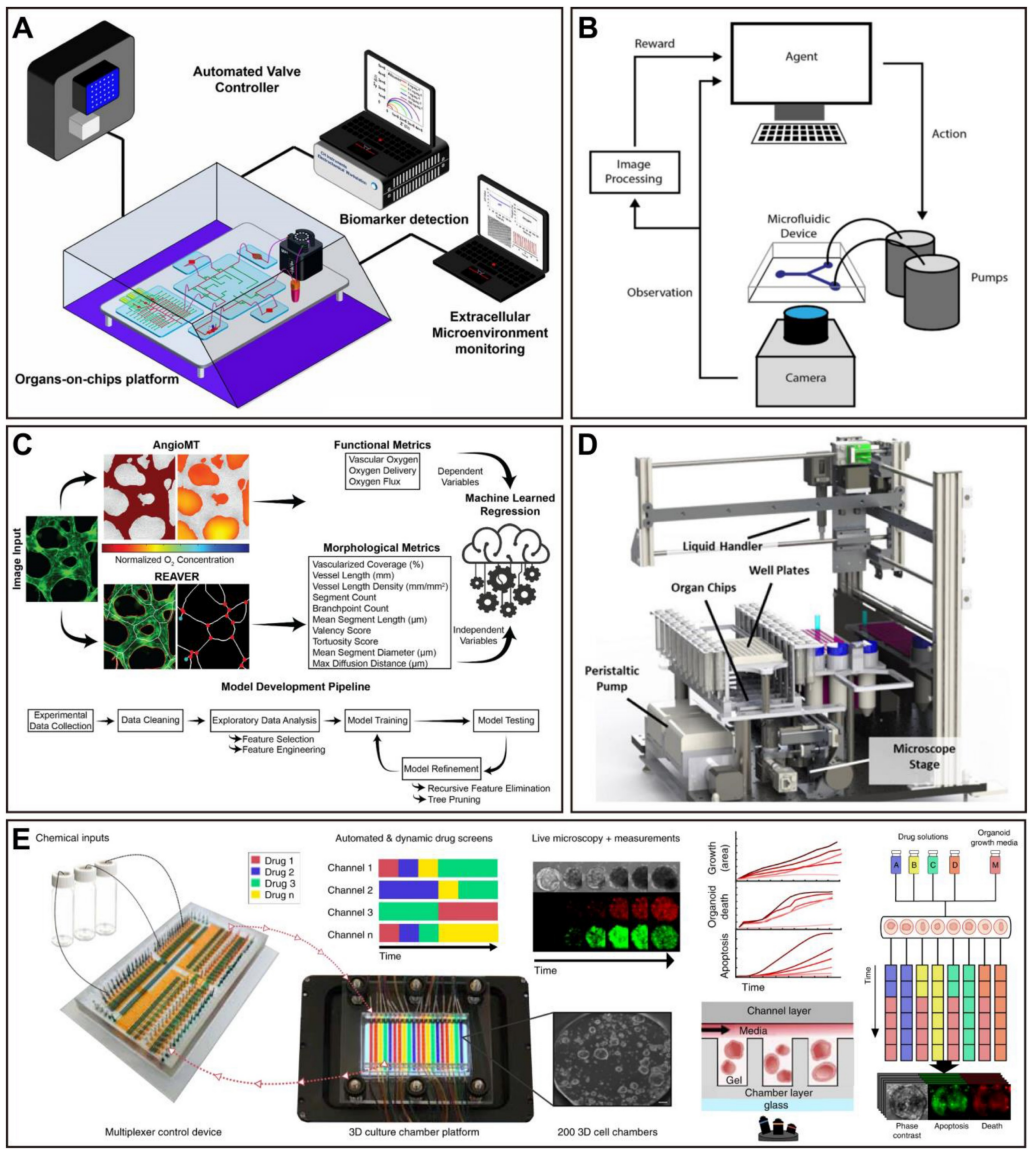
** Representative examples of OoCs combined with AI or automation for experimental design and control. (A)** A multi-OoC system includes a physical, biochemical, and optical sensing platform that operates organ units continuously, dynamically, and automatically, achieving *in situ* monitoring of organoid behaviors. Adapted with permission from 197, copyright 2017 National Academy of Sciences. **(B)** Automatically adjusts flow conditions with reinforcement learning to assist microfluidic platforms in maintaining stable flow conditions over time. Adapted with permission from 248, copyright 2018 American Chemical Society. **(C)** The utilization of supervised ML to evaluate the morphological and biological functions of vascularized OoCs. Adapted with permission from 249, copyright 2023 Springer International Publishing. **(D)** A robotic system consists of a 3-axis motion system, automated liquid handler, peristaltic pump, and custom microscope stage that enables the continuous perfusion, linking, and image analysis of up to ten organ models. Adapted with permission from 181, copyright 2020 Springer Nature. **(E)** A microfluidic platform based on a programmable membrane-valve allows to provision of combinatorial and dynamic drug therapies and enables analysis of organoids in real-time for personalized drug screening. Adapted with permission from 250, copyright 2020 Nature Publishing Group.

Except for microscopic images, various data types, including electrochemical detection data, can serve as inputs for DL model development for training, enabling the detection of organ functions and drug treatment endpoints on OoCs. Electrochemical monitoring technology is automated and noninvasive, making it suitable for long-term operation in the monitoring of OoCs. By integrating a variety of online physical and biological electrochemical sensors to accomplish continual, automated, and *in situ* sensing of microenvironment biophysical and biochemical parameters (Figure 9A) 197, noninvasive monitoring OoCs and performing real-time data analysis (e.g., measurement of nutrients, metabolites, proteins, growth factors, exosomes, shear forces, current/electrical resistance, pH, oxygen levels, and drug uptake) could be achieved for autonomous decision-making 240. Importantly, all these real-time and continuously collected data could be combined with AI-based data processing for studying and optimizing closed-loop feedback-based experimental parameters. Eventually, the system will be able to automatically regulate and control various functional parameters of OoCs, achieving the development of intelligent OoCs 62, 241.

### AI-based experimental design and control of OoCs

Although the most of current applications focus on post-experimental data analysis, DL has increasing potential for designing microfluidic systems and controlling systems during experiments 60. Microfluidics represents an excellent platform for supporting automation and intelligent control of reaction conditions 242. In the preparation phase of a project, DL can be employed for devices design and materials selection to make OoCs more suitable for specific applications 19. Traditionally, soft lithography, photolithography, and etching techniques have been widely utilized in the manufacture of OoC devices. However, these methods presented serious limitations that hinder the pace of development and innovation of microfluidic applications 243. Given this, 3D printing has the potential to be a promising solution. This technique provides high-throughput and scalability, and allows for industrial means of mass production. To date, a number of studies have applied 3D printing technology to build molds for manufacturing OoC devices. In a study, 3D printing was utilized in the manufacture of almost all chip components 244. To realize high precision microscale structures, material properties, and printing parameters require delicate control 245. During this process, AI could be applied to provide support to improve accuracy and efficiency, such as 1) the optimization of processes; 2) the detection of manufacturing defects; 3) the evaluation of dimensional accuracy; and 4) the prediction of material properties 246. For instance, a computer vision-based (CNN-aided calibration) approach was used to rapidly and precisely design microfluidic devices and minimize absolute errors in device manufacturing, which offers a convenient, effective, and efficient solution for 3D printing of OoC platforms 247.

During the experiment, reinforcement learning (RL)-based deep Q network can utilize image feedback to assist in retaining stable flow conditions for prolonged periods by automatically adjusting flow conditions to mitigate the inconsistent system performance exhibited in microfluidic platforms during extended experiments (Figure 9B) 248, which could be applied to the control of culture media in OoCs. An emerging direction in the development of OoCs is vascularization, which is becoming a significant and necessary physiological level feature of most OoCs 249. Recently, a study assessed the oxygen transport capacity of vascular network association with the most common morphological indicators through ML algorithms such as multiple linear regression and random forest. This approach will assist in measuring the performance or biological function of vascularized networks in OoCs (Figure 9C) 249. For lung OoCs, mechanical stretch is used to mimic the cyclical expansion, resulting in tissue mechanical force control being critical. Therefore, automatic control of the applied tissue mechanical forces can be achieved by DL of cell morphology and microenvironment. Importantly, DL allows for the real-time monitoring of the entire system performance while continuously detecting cell processes and biomarkers without harming cell viability 19. Researchers have proposed to regulate microenvironmental parameters through spectroscopy, automated multisensor, and microscopy monitoring systems, as well as through machine-intelligent data-driven optimization, as the cell microenvironment is crucial for maintaining physiologically relevant organ functions and responses to drugs 62. A multi-OoC system achieved exact control of flow distribution and drug distribution to different organs using an on-board pneumatically-driven pump with independently programmable flow rates 182. Furthermore, an automated microfluidic platform was developed to accomplish dynamic and combinatorial drug screening, which allows for highly dynamic, reproducible, and reliable analyses of patient-derived organoids (Figure 9E) 250. What's exciting is that liquid-handling robotics control systems allowed the automated culture, perfusion, medium addition, fluidic linking, sample collection, and *in situ* microscopy imaging of up to ten organ models (Figure 9D) 181, which will be easier to integrate with pharmaceutical robotic pipelines 73. Therefore, automation allows real-time data collection and analysis for feedback on target results. In the near future, it is conceivable that AI-guided organ-on-a-chip may in fact be more fundamentally natural than human control.

In conclusion, even though only a few OoC studies have incorporated AI, we believe that these existing studies are sufficient to indicate exciting prospects for synergy between OoCs and AI in future drug evaluation (Figure 6-9). Nevertheless, much follow-up work and collaboration are still needed to drive the development of the combination of OoCs and AI, and ultimately to contribute to the advancement in the field of drug evaluation.

## The future prospect of OoCs and AI in drug evaluation

### OoCs will become an indispensable part of the future drug evaluation system

OoCs using human cell sources (e.g., primary cells, cell lines, iPSCs, or organoids) could possibly eliminate the effects of cross-species differences introduced by utilizing animal models for clinical drug studies. A major obstacle of OoCs is the limited lifespan of cells in the device, and such limitation is exacerbated when not using immortalized cell lines 251. Despite the widespread view that cell lines typically lack the ability to simulate tissue-specific functions with high fidelity; however, cell lines are able to provide a practically limitless supply of similar cells that could be utilized for studies in higher-throughput. This could potentially improve the reproducibility of results, making it highly valuable for drug development in the early stages 73. A study demonstrated that the reproducibility was greatly dependent on the cell source 252. It is noteworthy to mention that iPSCs, while iPSCs provide the same advantages, they are frequently limited the wide applicability by their failure to display a fully mature differentiated phenotype, and the necessary purity for many tissues 73.

OoCs have been extensively used to build a variety of *in vitro* disease models, an important aspect of which is rare disease models, and OoCs could fill the gaps where animal models do not work or even do not exist. To date, only about 400 of over 7,000 identified rare diseases have active research programs due to the absence of animal models for others, resulting in significant hindrances to the development of new drugs in the field 253. In the past, OoCs have been used to successfully model many rare diseases. Recently, TNT005, a drug received clinical approval from the FDA based on preclinical efficacy data obtained from rare disease-on-a-chip, showing that OoCs have great potential in the field of rare diseases leading to the generation of IND 254. OoCs-based models for rare diseases have the potential to produce significant data that are typically not observable in *in vitro* and *in vivo* models or clinical samples, as OoCs enable long-term and real-time monitoring of changes in physiological processes. Through further analysis of these data by ML/DL in real-time, it is possible to analyze the progression at the molecular level of such diseases, and eventually discover the specific mechanisms by which the diseases occur 19. Thus, OoCs have a promising opportunity in rare diseases. Besides, similar approaches are applicable to other diseases as well. Furthermore, OoCs could be seeded with iPSCs and patient-derived organoids to develop patient-specific models, and deliver on the promise of advanced personalized medicine.

Perhaps more importantly, OoCs can be used first time in emergencies for disease mechanism research and drug repurposing, discovery, and toxicity evaluation. For instance, during the COVID-19 global pandemic, several OoC platforms have been successfully used 27, 29, 111, 155, 203, 255-258. A vascularized lung OoC platform was utilized for SARS-CoV-2 infection and to identify the virus-induced vascular damage, including inflammatory response and loss of barrier integrity, the latter can be alleviated by tocilizumab treatment 255. The same team also revealed the SARS-CoV-2-induced intestinal injury and immune responses with the use of a gut OoC platform 256. Moreover, lung airway OoCs have also been used for the reconstruction of clinically relevant influenza virus evolution 257. Meanwhile, OoCs have also been successfully used in the study of other viruses 259, 260. Thus, the development of antiviral drugs will continue to be a major focus of drug discovery in the post-COVID-19 era 261.

### New AI for new OoCs

AI itself is a rapidly developing discipline, and deep learning networks and automated machine learning have promoted the development of generative AI. AI has shown unprecedented creativity, based on OoCs' own needs and new creative needs, the development of OoCs will be greatly accelerated.

Firstly, AI can address the bottleneck issue in the development of OoCs hardware. PDMS is one of the most popular materials for fabricating microfluidic devices. However, there is a common concern that OoCs made out of PDMS are unable to be utilized efficiently for drug research because of drug absorption 262. But this issue has not proven to be as serious a concern as first thought, because only hydrophobic drugs are absorbed by PDMS, which is only a small portion of the drug development pipeline 73. In fact, as previously mentioned, those showing *in vivo* simulations in response to clinically relevant drug exposures, as well as OoC platforms capable of quantitatively predicting human pharmacokinetic parameters, were manufactured almost by PDMS (Table 1). Despite this, the absorption of PDMS remains a significant challenge for drug screening of hydrophobic compounds like small molecule drugs, which could result in biased experimental results. Now, researchers could avoid the risk of small molecule drugs being absorbed by using alternative materials such as inorganic (e.g., glass and silicon), elastomeric (e.g., polyesters and polyurethane), and thermoplastic (e.g., poly(methyl methacrylate) and polycarbonate) materials, or coating PDMS with non-absorbent coatings 31, 44, 263, 264. Nevertheless, a careful characterization of the adsorption/absorption curves is required for OoC platforms, regardless of the material of manufacture chosen 79. Furthermore, PDMS-based devices frequently need to be manually cast, punched, and assembled, thus significantly reducing the reproducibility and throughput of the fabrication process 265, and that could be addressed by 3D printing/bioprinting 266, 267. 3D bioprinting technology has been widely utilized in the construction of *in vitro* tissue/organ models and testing devices for drug screening 6, including OoCs. It makes it possible to precisely distribute cells or biomaterial in a target region, which allows the creation of more complex structures and microenvironments that more accurately mimic the function of living organisms 6, 268. In fact, in other fields, AI and 3D bioprinting have been widely combined. AI-assisted artificial design of structures has a much faster optimization iteration speed than engineers, and the identification and discovery of material defects based on AI feature recognition are also faster and more accurate. In the future, generative AI will bring deeper technological changes to the development of OoCs in terms of drawing design, structural optimization, and even full process production.

Despite this, OoCs remain facing various challenges that must get past in order to promote their physiological relevance and facilitate their translation into the clinic. For example, introducing more cell subtypes, metabolites, and microbiomes, as well as biochemical and biophysical gradients 175, or vascularization and innervation of organs 269, to increase the complexity of the models. More importantly, the body-on-a-chip system needs to be scaled according to the sizes of actual organs *in vivo*, relying on appropriate scaling rules and methods, and the fluid volume and dynamics in chips should be adjusted in accordance with specific human organs 175, which are key to simulate physiological responses. Of note, in the experiment design, a balance between the system's feasibility and complexity should be considered carefully 174. In addition, the use of robust culture media, especially for multi-OoCs, which can help to promote cell survival in different organ types and keep the various organs functional 175, is equally significant, as a key challenge. In microfluidic devices, organ function often declines in long-term culture, and tissue necrosis caused by a lack of oxygen diffusion continues to be a barrier to the use of larger or more complex organs, although vascularization of most organs has been achieved. In view of this, vascularized constructs with a tissue-specific vasculature and perfusable vascular network will offer the foundation for reliable OoCs with sustained functionality 269. Notably, AI can use reinforcement learning to identify optimal medium compositions and dynamic culture conditions for particular cell types, possibly extending the lifespan and functionality of present *in vitro* organ models 62. More importantly, for integrated multiorgan models, identifying optimal medium compositions and dynamic culture conditions for co-culture of various cell types, would make more sense.

Secondly, AI will increase the detection throughput of OoCs. Typically, OoC devices are low-throughput, which limits their applicability in the early phases of drug discovery 270. However, considering that there are multiple stages in drug discovery and development where OoCs could be implemented, more complex low-throughput to medium-throughput OoC devices may be more useful at later stages, such as drug efficacy studies 79. For pharmaceutical companies, the model can be selected according to the stage of drug development, after considering time, cost, and benefits. Although physiological relevance may be compromised in exchange for high-throughput 93, in fact, the recently developed high-throughput OoC devices still retain the key features required for drug developers 31, 203, 205. Yet, the increase of larger data due to the use of high-throughput platforms is a new challenge, this could probably be solved with AI, as previously discussed. In addition, automation is another key requirement for developing reliable and high-throughput platforms 271, while such devices have been developed, they are extremely rare 181. Importantly, OoCs successfully integrated with AI will be invaluable for its applications in drug development, and investigating how better to integrate is worthwhile.

Finally, AI further promotes the convenience of OoCs data processing. At present, the iterative development speed of AI is astonishing. A simpler, more open, and more user-friendly AI-based algorithm platform will be developed, making image and big data analysis for OoCs dynamic processes simpler. Recently, especially with the emergence of open AI, it will also be applied in the scientific field. The design of OoCs and the extraction of more biological-related features will be completed by AI. Imagine the future, where people will become emitters of commands, AI-controlled automated OoCs production and analysis platforms will present the data we need, and drug evaluation will become an easy task at that time.

We can foresee that AI will further help us invent new OoCs products in the design, production, and control of OoCs, as well as data processing, to expand the application of OoCs. While OoCs' potential is exciting, the technology is still in its early stage 79. Also, despite the significant benefits of coupling OoCs with AI, there are still some challenges ahead, especially as this fall within the healthcare field. For instance, as mentioned in section 5.2, how to achieve the proper explanation of the collected data. A typical issue of machine learning, particularly deep learning models is known as “black box approach”; that is, the lack of interpretability, which limits the obtaining of a suitable explanation from such models on how they arrive at their results 206. This lack of interpretability may significantly hinder their application in the short term. Thus, explainable models are necessary to be developed to improve trust, which will promote the application of models in OoCs. Meanwhile, repeatability is another important issue. It may produce different results by using different algorithms, thereby increasing uncertainty 206. To date, some solutions have been proposed to move AI towards reproducible 272. The quality of the model depends on the quality and characteristics of the data, so the datasets collection and selection are very significant for building a model, which directly impacts the accuracy and predictability of the model. Of note, training, validation, and testing datasets are crucial for model development, but the amounts of datasets required depends on the complexity of the data type and the task 93, 273. Despite such challenges, as both field progresses, the application of AI will undoubtedly bring greater vitality and impetus to the development of OoCs, especially in the area of drug evaluation.

Last but not the least, while OoCs have advanced significantly in the academic environment, and a few OoC platforms have even been successfully converted into commercial products, numerous challenges prevent its complete deployment in an industrial setting, and OoCs are still marginalized in the pharmaceutical industry 14, 174. To bridge the academia-to-industry gap and facilitate the adoption and implementation of OoCs in the drug development process, ongoing engagement, and discussions with OoCs developers, end users, and regulatory bodies are critical 79, 274. From a long-term viewpoint, as technology improves and cost decrease, OoCs will finally be better accepted and adopted by the pharmaceutical industry.

## Conclusion

In summary, organ-on-a-chip successfully replicates the critical physiological functions and environment of the human organs, as a state-of-the-art *in vitro* model, showing encouraging performances in a variety of drug evaluation platforms. Nevertheless, the current OoCs still must face many challenges that take it from academia to industry. Continuing development with AI, it is undeniable that OoCs will likely dramatically change drug development, disease modeling, and personalized medicine.

## Figures and Tables

**Table 1:** Representative examples of drug evaluation in single-organ-on-a-chip.

Single-organ	Materials	Channel	Cell sources	Applications	Ref.
Brain/BBB	PDMS	Two	Human brain microvascular endothelial cells (HBMVECs) (iPSCs), pericytes, astrocytes	Drug and antibody transport	23
	PDMS	Three	Microglia cells (HMC3), HBMVECs, astrocytes, pericytes	Stem cell therapy efficacy	24
	PDMS	Two	HBMVECs (iPSCs), brain pericytes, astrocytes	Drug transport	25
Lung	PDMS	Two	Human pulmonary microvascular endothelial cells (HPMECs), alveolar epithelial cells, neutrophils	Nanoparticulate toxicity	22
	PDMS	Two	HPMECs, alveolar epithelial cells	Drug toxicity	26
	PDMS	Two	Human lung bronchial-airway epithelial basal stem cells, HPMECs, neutrophils	Drug efficacy	27
Heart	PDMS and PMMA	-	Human umbilical vein endothelial cells (HUVECs) (3D printed), cardiomyocytes (iPSCs)	Drug toxicity	28
	PDMS	Two	Cardiomyocytes (iPSCs)	Drug toxicity	29
	Gelatin	Two	Cardiomyocytes (iPSCs)	Drug toxicity	30
Liver	Glass and plastic	Two	Hepatocytes (iPSCs), HMEC-1 endothelial cells, THP-1	Drug toxicity	31
	PDMS	Three	Hepatocytes, Kupffer cells, liver sinusoidal endothelial cells (HLSECs), hepatic stellate cells	Drug efficacy	32
	PDMS	Two	HLSECs, hepatocytes, stellate cells, Kupffer cells	Human and cross-species (rat, dog) drug toxicities	33
Kidney	PDMS	Two	Human proximal tubular epithelial cells	Drug transport and toxicity	34
	Plastic	Three	Podocytes, glomerular endothelial cells	Drug efficacy and toxicity	35
	PDMS	Two	Podocytes, vascular endothelial cells	Drug toxicity	36
Gut	PDMS	Two	Caco2	Drug permeability	37
	PDMS	Two	HUVECs, intestinal epithelial cells, Caco2	Drug efficacy	38
	PDMS	Two	Human intestinal microvascular endothelial cells, Caco2	Microbiome-host interactions	39
Vasculature	PDMS	Four	HUVECs, lung fibroblasts	Nanomedicine efficacy	40
	PDMS	Two	HUVECs	mAb therapy toxicity	41
	PDMS	Three	HUVECs	Drug efficacy	42
Skin	PDMS	One	Fibroblasts, keratinocytes	Drug efficacy	43
	PMMA	Two	Keratinocytes	Drug toxicity	44
Bone/Bone barrow	PDMS	Two	Bone marrow stromal cells, HUVECs, CD34^+^ cells	Drug toxicity	45
	PDMS	Five	Bone marrow mesenchymal stem cells, HUVECs, CD34^+^ cells	Radiation toxicity	46
Retina	PDMS	Two	Retinal pigmented epithelial cells, seven essential retinal cells (iPSCs)	Drug toxicity	47
	PDMS	Four	Retinal pigment epithelium cells (ARPE-19), HUVECs, lung fibroblasts	mAb therapy efficacy	48
Muscle	PDMS	Three	Human aortic smooth muscle cells (HAoSMCs)	Drug efficacy	49
	PDMS	-	HAoSMCs	Drug efficacy	50
Fat	PDMS	-	Adipocytes, peripheral blood mononuclear cells (PBMCs)	Drug efficacy, cell-cell interaction	51
	PDMS	Two	Adipocytes	Drug efficacy	52
Tumor/Cancer	PDMS	Two	Human lung microvascular endothelial cells, lung alveolar epithelial cells, non-small-cell lung cancer cell line (H1975)	Drug efficacy	53
	PDMS	Three	HBMVECs, microglia cells (HMC3 or patients), PBMCs, macrophages	Immunotherapy efficacy	54
	PDMS	Two	Human colonic microvascular endothelial cells (HCoMECs), colorectal cancer cell line (HCT-116)	Nanomedicine delivery	55
	PDMS	Two	Human gastric epithelial cells (NCI-N87)	Drug efficacy	56
Spinal	Plastic	-	Human embryonic stem cells (WA09)	Drug efficacy	275
Cartilage	PDMS	Two	HUVECs, synovial fibroblasts, articular chondrocytes, monocytes, synovial fluid	Drug efficacy	276
Placenta	PDMS	Two	Human placental villous endothelial cells (HPVECs), trophoblast cells (BeWo b30)	Drug transport	277
Pancreas	PDMS and glass	Two	Pancreatic ductal epithelial cells (PDECs), pancreatic islets (all patient)	Disease modeling	278
Teeth	PDMS	Three	Stem cells from the apical papilla (SCAPs), dentinal tubules	Biomaterials toxicity	279
Uterus	PDMS	Five	HUVECs, endometrial epithelial cells, endometrial stromal fibroblasts	Drug efficacy	280
Vagina	PDMS	Two	Human vaginal epithelial cells, uterine fibroblasts	Microbiome-host interactions	82

**Table 2:** Representative examples of multi-organ-on-a-chip for applications in drug evaluation.

Number	Multi-organ	Cell types	Medium	Duration	Applications	Ref.
Two	Liver-lung	Primary cell, cell line	PneumaCult™-ALI	28d	Drug toxicity	169
	Liver-heart	Primary cell, iPSCs	Serum-free medium (HSL2 and HLS3)	28d	Drug toxicity	170
	Liver-skin	EpiDerm^TM^, primary cell, cell line	“Co-culture Medium”: EPI-100-NMM-WE	6d	Drug PK/PD analysis	179
	Live-heart	Primary cell, iPSCs	RPMI 1640 and DMEM (1:1 ratio)	5d	Drug toxicity	197
	Lung-skin	Primary cell, cell line	E3 medium supplemented with glucose	5d	mAb therapy efficacy and toxicity	198
	Liver-gut	Primary cell, cell line	Serum-free common medium contained Williams E medium, Gibco Cocktail B, and hydrocortisone	3d	Drug PK modeling	281
	Liver-testis	Primary cell, cell line	William's medium E supplemented with CTS^TM^ KnockOut^TM^ SR XenoFree medium	7d	Drug toxicity	282
	Liver-gut/skin	Primary cell, cell line, tissue	N.A	14d	Oral or transdermal drug absorption	283
	Liver-pancreas	iPSCs	Co‐culture medium: RPMI 1640 with glucose, N-acetylcysteine, B27 supplement, N2 supplement, GlutaMAX, and non‐essential amino acids	30d	Glucose‐stimulated insulin secretion, drug efficacy	183
	Liver-kidney	Cell line	DMEM (high glucose)	1d	Drug metabolism	284
Three	Liver-kidney-gut/bone marrow	Primary cell, cell line	“Blood substitute”: DMEM/F12 with EGM-2 supplements, growth factors, and FBS	10d	Drug PK/PD analysis and toxicity	177
	Liver-kidney-gut	Cell line	DMEM (high glucose)	3d	Drug PK analysis, and metabolism	178
	Liver-lung-heart	Primary cell, iPSCs	α-MEM with FBS and L-glutamine	9d	Drug efficacy, toxicity, and metabolism	285
	Liver-heart-skeletal muscle	Primary cell, iPSCs	Serum-free medium (blood surrogate)	7d	Drug PK/PD analysis, immune response	286
	Liver-lung-colon cancer	Cell line	DMEM-10 and EGM-2 with FBS (3:1 ratio)	15d	Cancer metastasis	287
	Liver-lung-breast cancer	Cell line	“Device medium”: EMEM supplement with FBS	2d	Inhalation and intravenous therapy, drug efficacy and toxicity	288
	Liver-lung-gut	Cell line	DMEM supplement with FBS and MEM non-essential amino acids	3d	Oral administration, drug efficacy	289
Four	Liver-heart-neuronal-muscle	Primary cell, iPSCs, stem cell, cell line	Serum-free medium supplemented with growth factors	14d	Drug toxicity	172
	Liver-gut-colon cancer-connective tissue	Cell line	Medium 670	3d	Drug metabolism and efficacy	290
	Liver-kidney-gut-brain	iPSCs	HepaRG medium	14d	Personalized medicine	199
	Liver-kidney-BBB-gut	Primary cell, cell line, iPSCs	Functional coupling medium	N.A	Drug metabolism and PK analysis	291
	Liver-heart-breast-vulva cancer	Primary cell, cell line, iPSCs	Custom serum-free medium formulation	14d	Drug metabolism, efficacy, and toxicity	292
Five	Liver-fallopian tube-uterine-cervix-ovary (mouse)	Primary cell	Maturation medium (with prolactin, day 0 to day 14)	28d	Human menstrual cycle	173
Six	Liver-heart-lung- vasculature-testis-brain/colon (rabbit)	Primary cell, iPSCs, cell line, stromal mesenchymal cell, stem cell	Testis organoid media and EGM media (with supplements, without FBS) (1:1 ratio)	28d	Drug toxicity	293
	Liver-heart-lung- vasculature-brain-testis	Primary cell, iPSCs, stem cell	Testis organoid media and EGM media (with supplements, without FBS) (1:1 ratio)	21d	Drug metabolism and toxicity	294
Seven	Liver-brain-pancreas-lung-heart-gut-endometrium	Primary cell, cell line	N.A	14d	Drug toxicity	295
Eight	Liver-intestine-lung-brain-heart-skin-kidney-BBB	Primary cell, iPSCs, cell line	DMEM/F12 with EGM-2 supplements, FBS, and growth factors	21d	Drug PK analysis	181
Ten	Liver-intestine-lung-endometriumbrain-heart-pancreas (rat)-skin-kidney-muscle	Primary cell, iPSCs, cell line, tissue construct	Mixed medium	28d	Drug PK analysis	182
